# Formulation Development of a Multivalent Bioconjugate ExPEC Vaccine Candidate: Linking Early Design to Late-Stage Stability and Manufacturability

**DOI:** 10.3390/vaccines14080690

**Published:** 2026-08-11

**Authors:** Milena Opacic, Olga Labovitiadi, Paul de Goede, Martinus A.H. Capelle

**Affiliations:** Johnson & Johnson, 2333 CN Leiden, The Netherlands; olabovit@its.jnj.com (O.L.); pdegoede@its.jnj.com (P.d.G.);

**Keywords:** ExPEC, vaccine, bioconjugate, multivalency, formulation development, primary container, compatibility, stability, platform

## Abstract

Background: ExPEC9V was a 9-valent vaccine candidate intended for the prevention of invasive extraintestinal pathogenic *Escherichia coli* (ExPEC) disease (IED). Here, we describe more than a decade-long formulation development trajectory of this vaccine candidate aimed at establishing a stable, robust and scalable drug product that maintains long-term stability while addressing potential manufacturing challenges and increasing the probability of successful global deployment. Methods: Selected formulation development studies of the ExPEC multivalent vaccine candidate are summarized, spanning formulation screening, confirmation, and design of experiments (DoE)-based robustness, stability and compatibility studies. A formulation initially developed for an early low-valency vaccine candidate was subsequently tested and confirmed for candidates with additional serotypes incorporated based on antigen heterogeneity evidence. Contact materials employed included primary packaging—polycarbonate (PC) and polyethylene terephthalate glycol (PETG) bottles, borosilicate glass vials, stoppers, and prefilled syringes; vessel types—bags and stainless steel vessels used in drug substance (DS) and drug product (DP) manufacturing; and varying concentrations of tungsten and hydrogen peroxide. An evolving analytical panel was applied to assess attributes such as purity, protein concentration and degree of O-acetylation. Results: A phosphate-based formulation containing sorbitol, methionine, and polysorbate 80 showed superior stability in screening and was confirmed as fit for purpose across increasing vaccine valency. The ExPEC 9V drug product displayed remarkable thermal and formulation robustness, long-term (3 years) stability at 2–8 °C in glass vials and prefilled syringes, and compatibility with assessed primary containers and manufacturing materials. DoE-based robustness studies defined acceptable excipient and pH ranges, supporting a wide formulation design space. Conclusions: The development trajectory of the ExPEC9V vaccine candidate demonstrates that early prioritization of a robust, scalable formulation that remains fit for purpose across valency evolution supports a stable late-stage manufacturable drug product.

## 1. Introduction

Antimicrobial resistance (AMR) is considered a global health crisis, representing an increasing risk to human health, with current predictions forecasting 10 million deaths annually worldwide by 2050 [1,2,3,4]. Extraintestinal pathogenic *E. coli* (ExPEC) strains, which move from the gastrointestinal tract to extraintestinal locations, cause invasive ExPEC disease (IED), including sepsis, bacteremia (blood infections) and urinary tract infections. They are among the most threatening pathogens, particularly impacting immunocompromised individuals and adults aged 60 and older [5,6,7]. The World Health Organization (WHO) has prioritized AMR for research, highlighting the urgent need for effective treatments and vaccines to potentially prevent multidrug-resistant infections [8]. An increasing number of *E. coli* strains and their rising resistance to a variety of antibiotics make the effective treatment of many common infections more challenging, causing growing rates of hospital admissions and fatalities, as well as increasing healthcare costs [9]. This further emphasizes an urgent need for an effective vaccine that has the potential to prevent IED, including instances caused by drug-resistant *E. coli* strains.

**Figure 1:**
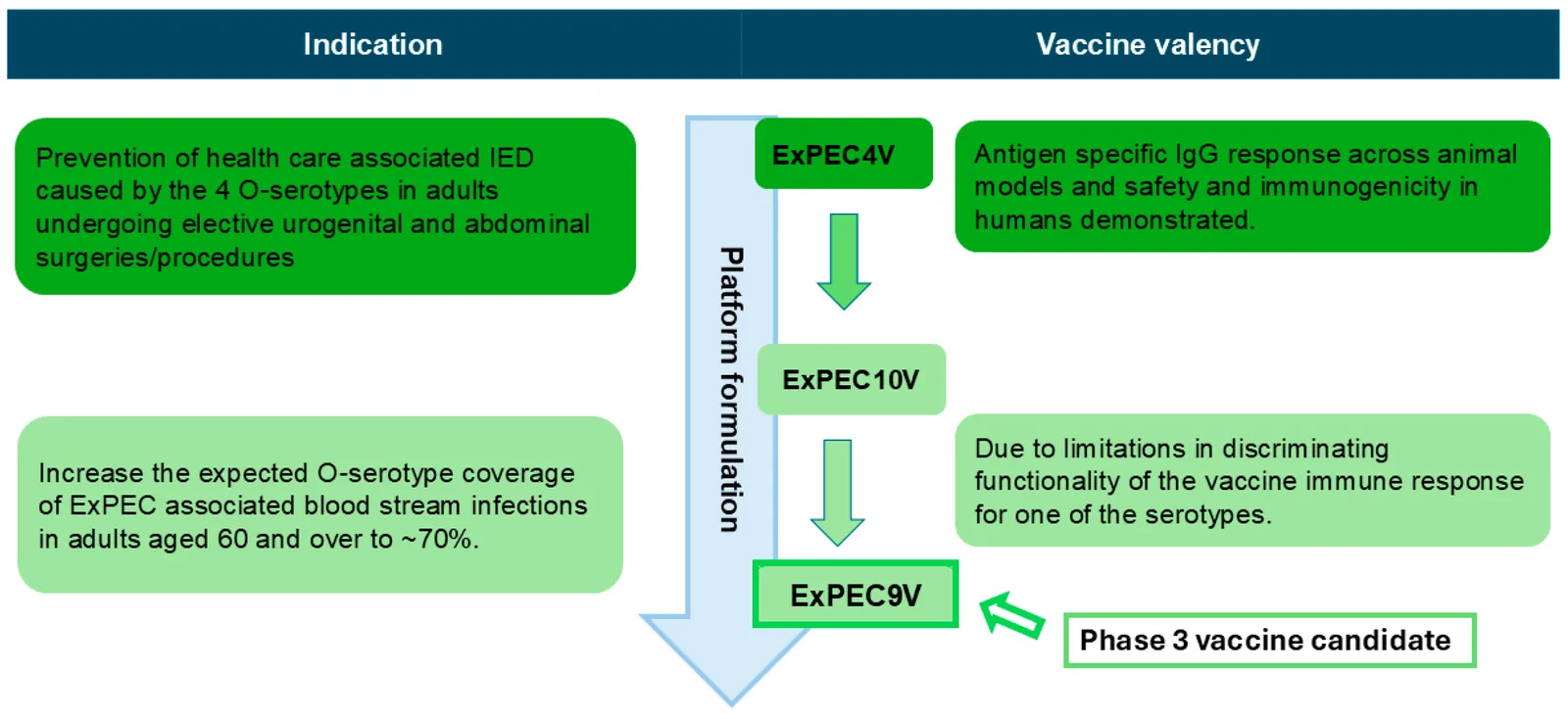
Evolution of ExPEC vaccine indication and valency. The composition of the ExPEC vaccine candidates is presented in Appendix A.1. References: Indication evolution [7,9,11,12,13]; antigen-specific IgG response [14]; safety in humans [1,11]; additional clinical data published on the Phase 1/2a and the Ph3 E.ngage [15,10].

Johnson and Johnson was developing an ExPEC vaccine candidate aimed at addressing this unmet need [6,9,10]. A platform approach was adopted, where a formulation was first developed for a low-valency vaccine candidate and then confirmed for ExPEC candidates containing additional serotypes included to overcome antigen heterogeneity, as informed by epidemiology. The structural similarity between the various serotypes was the driver for this approach and was confirmed during the course of our development work. The evolution of ExPEC vaccine valency and indication is presented in Figure 1 [11].

A Phase 3 candidate, ExPEC9V, was designed as a multivalent developmental prophylactic glycoconjugate vaccine consisting of the O-antigen polysaccharides of the extraintestinal pathogenic *E. coli* serotypes (Table A1) separately bioconjugated to a genetically detoxified form of 66 kDa exotoxin A (dEPA) carrier protein derived from *Pseudomonas aeruginosa* [10,16]. Previous ExPEC vaccine candidates were composed of the same dEPA protein with a different number of O-antigen polysaccharides (Figure 1). The bioconjugation process used for generating polysaccharide/protein complexes of the ExPEC vaccine candidate is described in Appendix A.2.

To ensure (glycoconjugate) vaccine stability during its (long-term) storage and shipment, it is crucial to optimize excipient selection, including buffering species and pH, as well as excipient concentrations [e.g., [17,18,19,20,21]. Commonly used classes of vaccine excipients are tonicity modifiers (e.g., salts and sugars), antioxidants (e.g., EDTA, vitamins C and E, and methionine), stabilizers (e.g., sugars, amino acids and proteins) as well as surfactants (e.g., polysorbate 20 and 80 and poloxamer 188 (P188).

Currently available licensed vaccines based on glycoconjugate technology are Haemophilus influenzae type b vaccines such as ActHIB and Hiberix, which use PRP conjugated to carriers like CRM197 or tetanus toxoid (TT); meningococcal vaccines such as Menactra and MENVEO employing serogroup-specific polysaccharides conjugated to CRM197 or diphtheria toxoid (DT); and pneumococcal vaccines such as Prevnar 13/20, VAXNEUVANCE and Synflorix that combine multiple serotype glycans with carriers including CRM197, TT, and Protein D [22,23,24,25]. As reflected in their regulatory product information (EPAR) they demonstrate multi-year labeled shelf lives under refrigerated storage (2–8 °C), while stability at 25 °C data can be limited and is product-specific [26,27,28,29,30].

However, these sources primarily describe labeled conditions rather than the experimental pathway that established formulation choices, including how formulations were screened, selected, stress-qualified, and made manufacturing- and container-robust, particularly as product complexity (e.g., valency) evolves.

In parallel, numerous glycoconjugate vaccine candidates, including biosynthetic and bioconjugate programs using rEPA/EPA-derived carrier proteins and O-antigens—across pathogens including *E. coli* multidrug-resistant sequence type 131 (MDR ST131), *Shigella dysenteriae* type 1, *Shigella flexneri 2a*, *Francisella tularensis*, *Klebsiella pneumoniae* and *Salmonella Typhi* [31,32]. Published literature has primarily emphasized antigen selection, carrier protein selection, conjugation or glycoengineering methodology, analytical characterization, immunogenicity, and protective efficacy across multiple pathogens [31,32,33,34,35,36,37,38,39,40,41,42,43]. By contrast, explicit drug-product formulation development and storage-stability datasets are limited. For *E. coli* MDR ST131, “O25B-EPA” and *Shigella flexneri 2a*, “Flexyn2a” are among the few EPA-based bioconjugates for which the clinical formulation composition is clearly disclosed: O25B-EPA and Flexyn2a drug products are formulated in Tris-buffered saline containing 25 mM Tris, 137 mM NaCl, and 2.7 mM KCl at pH 7.4 [31,34]. However, available *Shigella* EPA-bioconjugate clinical literature does not report systematic excipient screening, long-term real-time stability, stored adjuvant-antigen compatibility, container-closure compatibility, or explicit shelf-life justification. Overall, even though formulations of some of the bioconjugates have been discussed in a clinical context, based on the literature identified here, integrated datasets linking excipient selection, formulation optimization, late-stage compatibility, robustness, and multi-year stability remain sparse for O-antigen EPA/rEPA-based bioconjugates.

**Figure 2:**
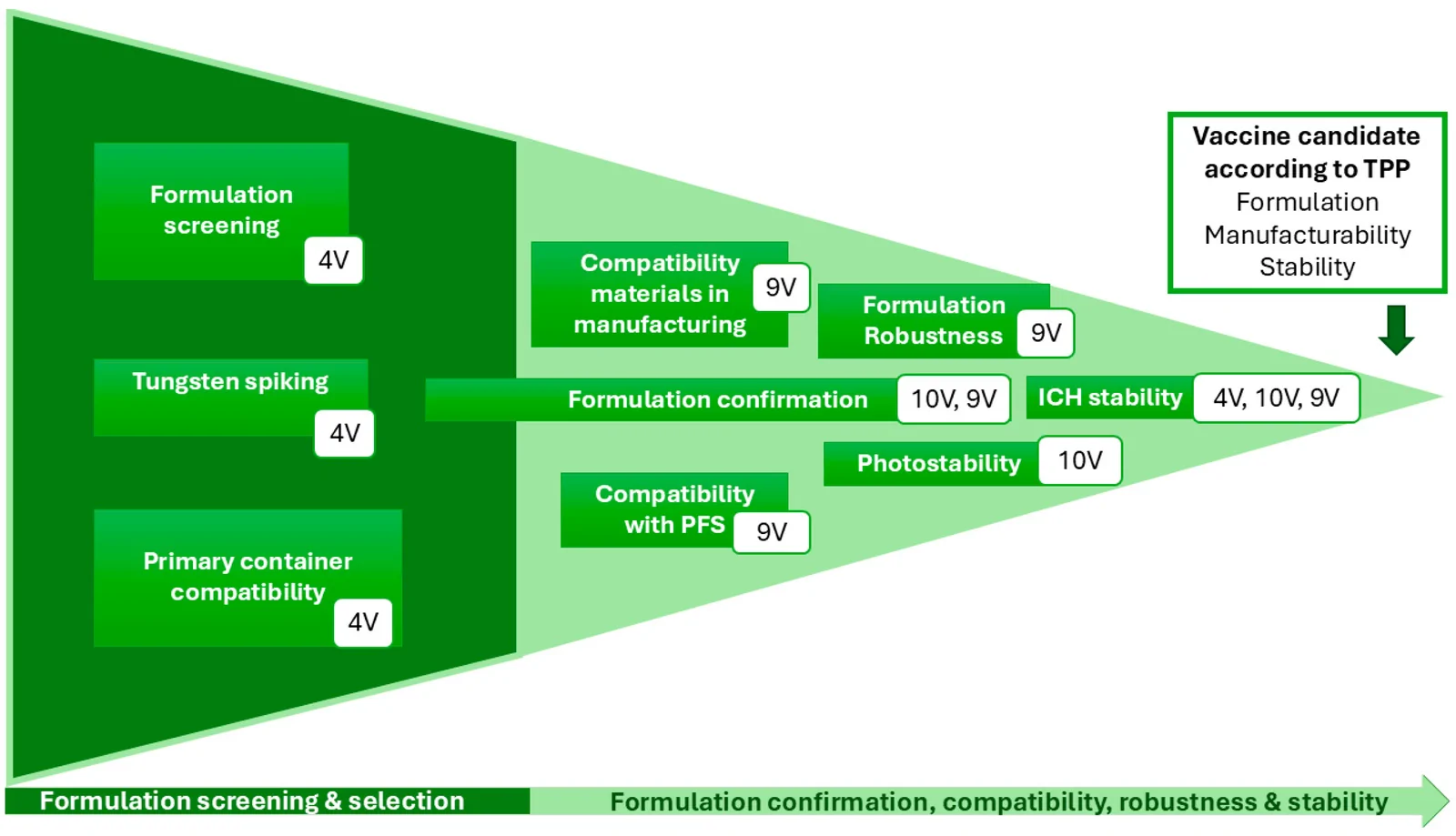
Summary of selected studies performed during the formulation development of the ExPEC multivalent vaccine.

Despite the discontinuation of the ExPEC9V clinical development during the Phase 3 E.mbrace study [9], multiple learnings were obtained that can benefit other (bio)conjugate vaccine development projects. Therefore, we here present a >10-year development trajectory for a dEPA-based multivalent ExPEC bioconjugate vaccine candidate, spanning formulation screening, formulation confirmation across valency evolution (4V→10V→9V), DoE-based robustness, and compatibility across assessed primary containers and manufacturing contact materials (Figure 2).

This work is supported by multi-year real-time stability data. An evolving analytical panel (e.g., SE-HPLC for glycoform distribution and aggregate monitoring, SoloVPE for protein quantification, and O-acetylation assays) is used to link early formulation screening to later-stage stability and compatibility studies.

## 2. Materials and Methods

### 2.1. Summary of the Formulation Development Trajectory

A summary of selected formulation-development studies (including the ICH stability study) of the ExPEC multivalent vaccine discussed in this manuscript is shown in Figure 2. Polysaccharide (PS) and EPA protein concentrations, including the PS/protein ratio for the selected DS and DP vaccine candidates discussed in this manuscript, are presented in Appendix A.1. Materials employed in the studies are detailed in Table A3. Contact materials included primary packaging (polycarbonate (PC) and polyethylene terephthalate glycol (PETG) bottles, borosilicate glass vials, stoppers, and prefilled syringes), vessel types (bags and stainless steel vessels) used in DS/DP manufacturing, and varying concentrations of tungsten and hydrogen peroxide. An overview of methods used per study and assessed attributes is presented in Table A5.

#### Studied Formulations

In total, more than 50 formulations were evaluated during this work. An initial screening focused on buffer species and pH, assessing >20 formulations containing sodium acetate, sodium succinate, histidine, Tris, or potassium phosphate across a pH range of 5–8. Based on the preliminary stability data, the best-performing histidine, phosphate and Tris-based formulations (30 candidate formulations) were progressed into formulation screening studies (Table A2) with ExPEC4V DP (serotype composition presented in Table A1) and the monovalent EO25 at concentrations representative of the drug substance (DS). The EO25 DS was selected as illustrative of the DS material, as it was shown to be one of the less stable DS serotypes and because it reflects DS serotypes with high relative mono- and diglycosylated percentages, has a polysaccharide (PS) content in the higher range compared with other DS serotypes, and contains partly acetylated rhamnose OH groups, which is an additional indicator of stability. Formulations comprising different buffer species, pH values, tonicity modifiers, antioxidants, and other excipients were investigated according to the formulation matrix in Table A2. All DS formulations were filled into PC (Thermo Scientific, Waltham, MA, USA) or PETG (Thermo Scientific) bottles. All DP formulations were filled into 2R Type I borosilicate glass vials (Schott, Mainz, Germany) and stoppered with 4432/50 chlorobutyl stoppers with Flurotec coating (West, Exton, PA, USA).

### 2.2. Stability and Compatibility of the Selected ExPEC Formulation

ExPEC DS and DP in the selected formulation were further assessed in multiple studies evaluating their stability and compatibility with different contact materials used during manufacturing as well as primary packaging. Tested contact materials included DP primary packaging—2R Type I borosilicate glass vials (OMPI), with a 4432/50 chlorobutyl stopper with Flurotec coating (West); and 1.0 mL short pre-filled syringes with either Nipro glass (OMPI) or Fiolax glass (BD), both containing an FM30 tip cap and a 4432/50 chlorobutyl stopper with Flurotec coating (West); FM30 PFS tip cap (Datwyler, Altdorf, Switzerland); and 366 dimethicone (silicone oil; DOW Corning, Midland, MI, USA).

Conditions and contact materials evaluated during the formulation development trajectory are summarized in Table A3 and Table A4, and an extensive list of attributes evaluated in these studies, including appearance and clarity, purity, protein content, and O-acetylation, along with the methods used, is presented in Table A5.

#### Stability and Compatibility Testing Conditions

To evaluate the ExPEC multivalent vaccine candidate stability during and after intended real-time storage, and when exposed to different stresses, the formulations evaluated were subjected to various conditions, including agitation, freeze/thaw, UV and visible light exposure and (developmental and ICH) stability testing on different temperature settings up to 40 °C (testing conditions are presented in Table A4, and attributes assessed and methods used in Table A5).

### 2.3. ExPEC9V Formulation Robustness Study, DoE

To demonstrate the robustness of the selected formulation, the ExPEC9V DP robustness study varied serotype concentration, excipient concentrations, pH and primary packaging as per Table 1. The study design was set according to design of experiments (DoE) methodology and comprised 45 sample formulations and 2 primary packaging types (2R Type I borosilicate glass vials (OMPI), 4432/50 chlorobutyl stopper with Flurotec coating (West); 1.0 mL short Fiolax glass PFS with a 4432/50 chlorobutyl stopper with Flurotec coating and FM30 tip cap (OMPI)). The study design is further detailed in Appendix E.

## 3. Results and Discussion

### 3.1. Formulation

#### 3.1.1. Formulation Screening

**Figure 3:**
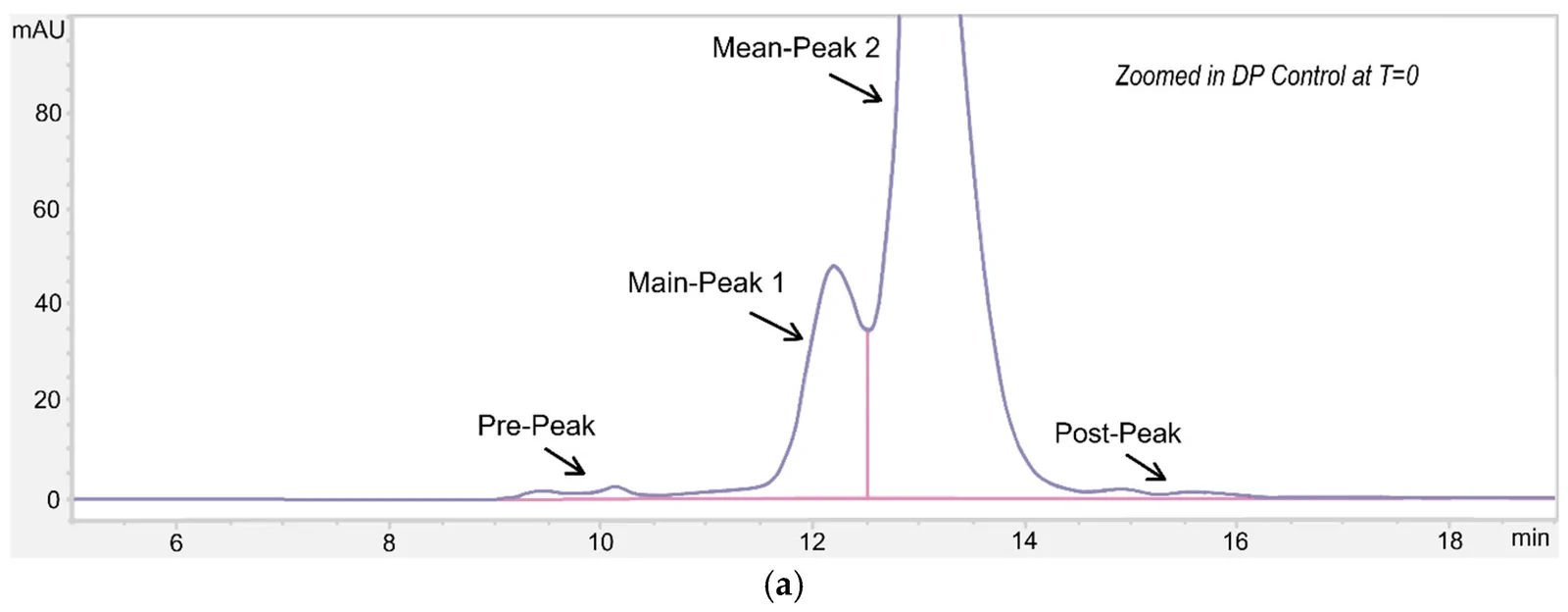

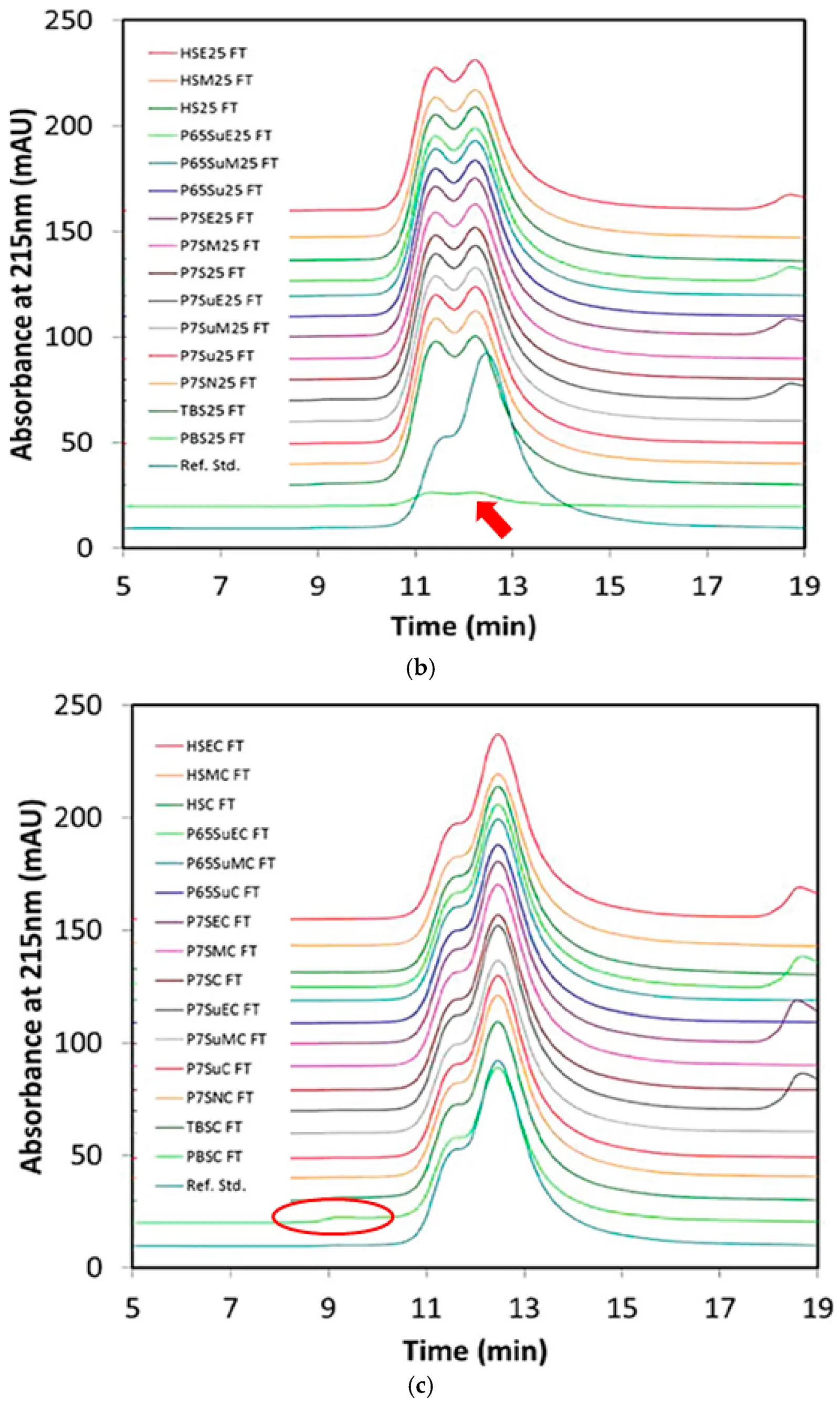
(**a**) An example of SEC-HPLC chromatogram peak integration of ExPEC formulation (ExPEC4V) showing the integrated regions: Pre-peak (earlier-eluting, higher apparent molecular weight species associated with aggregates); main peaks (intended bioconjugate glycoform distribution); and post-peak (later-eluting, lower apparent molecular weight species associated to degradation fragments). (**b**) SE-HPLC results for EO25 DS formulations. Red arrow: PBS25 DS formulation. (**c**) SE-HPLC results for ExPEC4V DP formulations (freeze/thaw). SE-HPLC peak percentage values for ExPEC4V DP and DS formulations exposed to freeze/thaw stress are presented in Table S5. Designations used: Histidine (H); K/Na phosphate (P); Tris buffered saline (TBS); phosphate buffered saline (PBS); pH (in case of phosphate formulations) = 6.5 (65) 7.0 (7), 7.5 (75); sorbitol (S); sucrose (Su); EDTA (E); methionine (M); ExPEC4V DP (C); EO25 DS (25). Red circle: PBSC DP formulation.

At this stage, a total of 30 formulations were assessed: 15 for the ExPEC multivalent (4V) DP and 15 for the ExPEC DS (Table A2). Stress conditions, including agitation stress, 5 freeze/thaw cycles, and extended storage at 40 °C were essential for discriminating between the screened formulations. As an illustration, formulations exposed to 5 freeze/thaw cycles are presented in Figure 3 (data listed in Table S4; an example chromatogram showing ExPEC4V peak integration is presented in Figure 3a). The PBS25 DS formulation was unstable after exposure to 5 consecutive freeze/thaw cycles (Figure 3b, indicated by the red arrow, Table S4), along with the PBSC DP and TBSC DP formulations, as demonstrated by increases in SE-HPLC pre-peak species (e.g. +1.6 % PBSC, and +28.5% PBS25) (Figure 3c, PBSC DP indicated by the red circle, Table S4). Notably, these three formulations did not have a cryoprotectant in their formulation composition, which likely explains their underperformance under freeze/thaw cycling stress compared to formulations that did contain sugar as a cryoprotectant. All other assessed EO25 DS and ExPEC4V DP formulations were not susceptible to aggregation after exposure to freeze/thaw stress (Figure 3b,c, Table S4). Other discriminatory stress conditions are described in detail in Appendix C. None of the formulations was impacted by storage at 5 °C for 12 weeks (Table S5).

After the first screening, three formulations were selected for both multivalent DP and DS: two leading phosphate formulations (P7SuEC and P7SMC) and a TBS formulation, and further assessed for the impact of additional stressors and compatibility with primary packaging (detailed in Appendix C.2 and Section 3.2.1).

**Figure 4:**
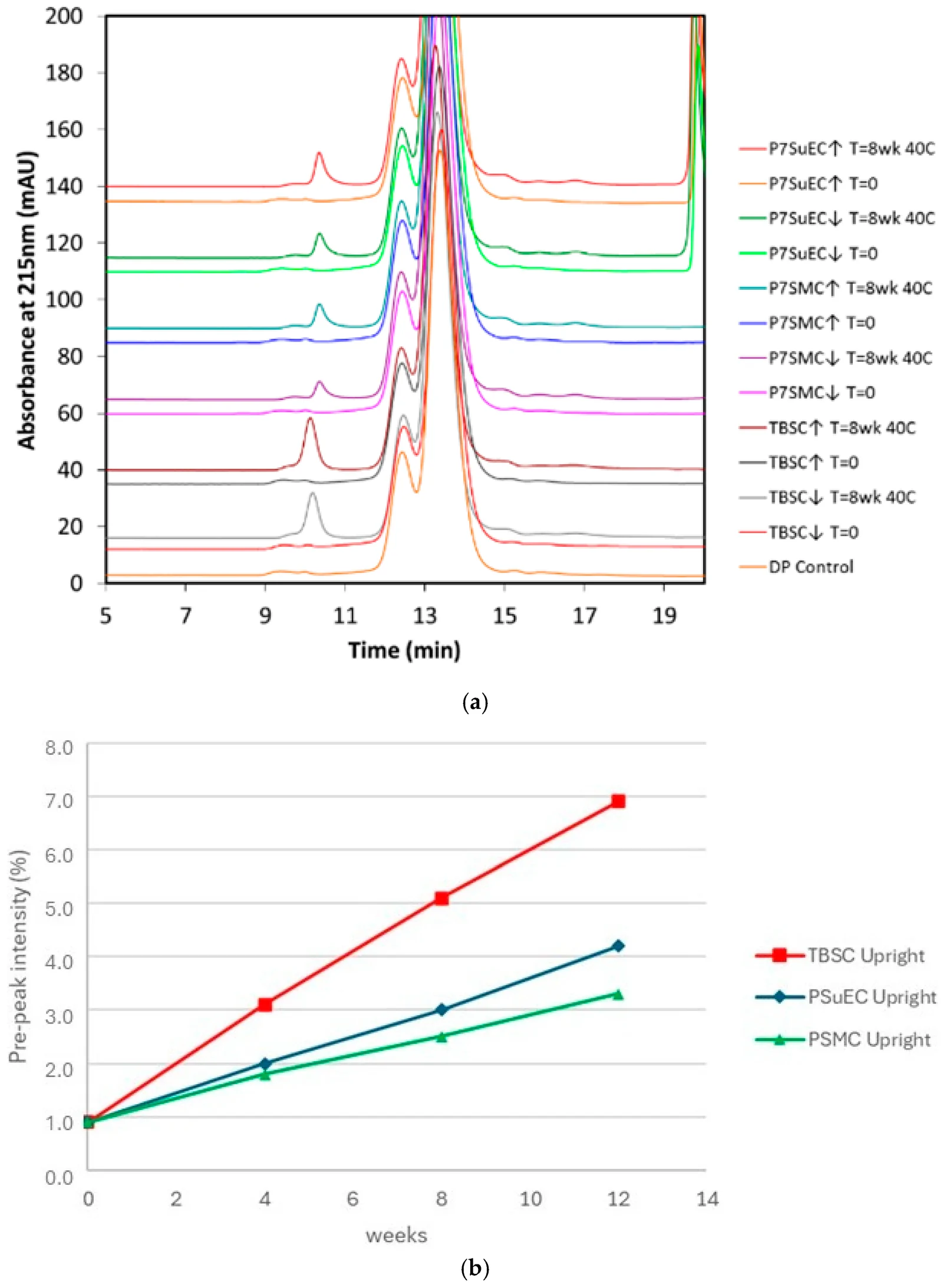
(**a**) SE-HPLC peak percentages of ExPEC DP formulations after 8 weeks of storage at 40 °C (↑: upright, ↓: inverted). (**b**) SE-HPLC pre-peak percentages of ExPEC DP formulations after storage at 40 °C for 12 weeks. Designations used: Histidine (H); K/Na phosphate (P); Tris buffered saline (TBS); phosphate buffered saline (PBS); pH (in the case of phosphate formulations) = 6.5 (65) 7.0 (7), 7.5 (75); sorbitol (S); sucrose (Su); EDTA (E); methionine (M); ExPEC4V DP (C); EO25 DS (25).

ExPEC DP and DS phosphate and TBS formulations exhibited comparable behavior when stored at 5 °C and 25 °C, while at 40 °C, phosphate formulations demonstrated superior stability independent of vial orientation. ExPEC4V DP showed this most clearly via reduced pre-peak growth by SE-HPLC (Figure 4a, Table S8). Among phosphate formulations, the sorbitol-containing PSMC had the best stability, with the least aggregation over 12 weeks at 40 °C (Figure 4b).

**Figure 5:**
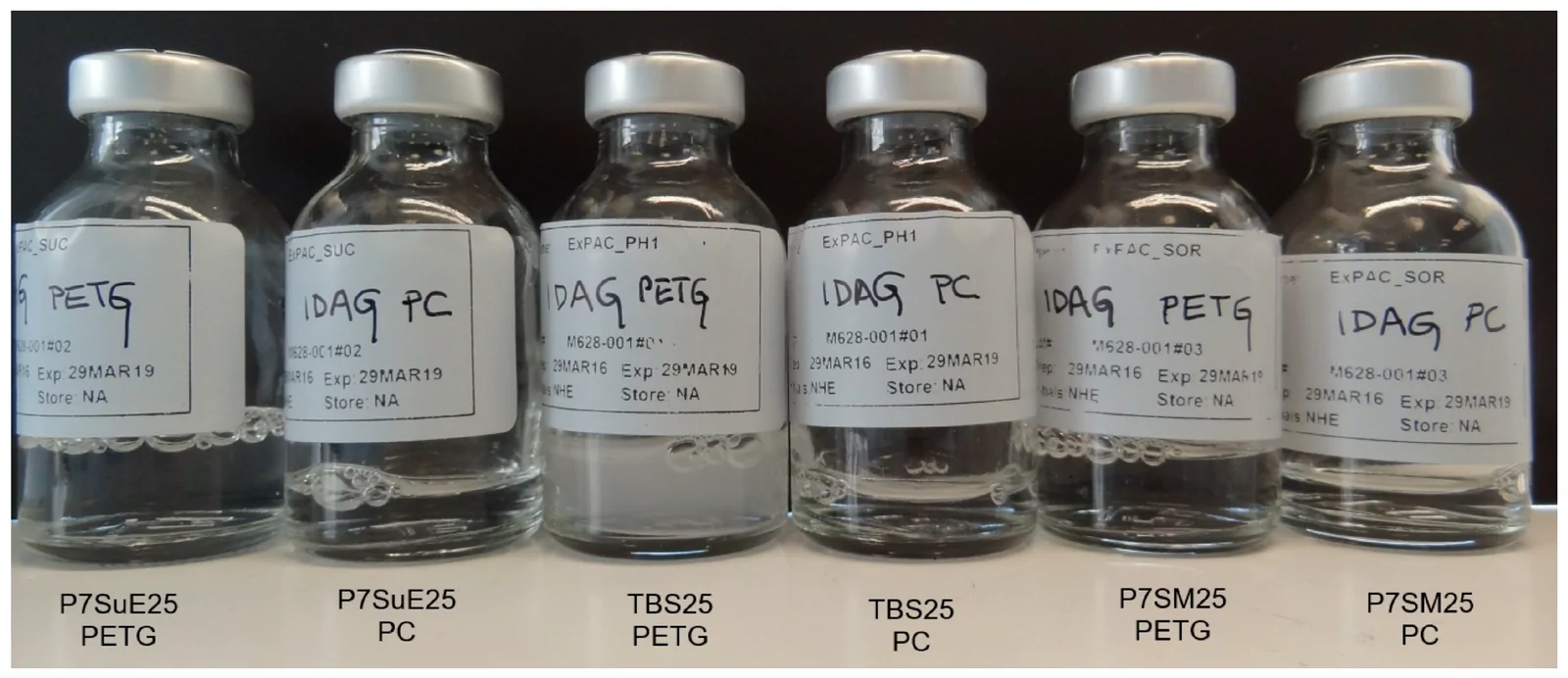
Visual appearance of three lead candidate EO25 formulations after 24 h of agitation at RT in polycarbonate (PC) and polyethylene terephthalate glycol (PETG) bottles. Note: After incubation for 24 h at RT, samples were poured in glass bottles to facilitate visual inspection. Designations used: K/Na phosphate (P); Tris buffered saline (TBS); phosphate buffered saline (PBS); pH (in case of phosphate formulations) = 6.5 (65) 7.0 (7), 7.5 (75); sorbitol (S); sucrose (Su); EDTA (E); methionine (M); EO25 DS (25).

In addition, enhanced stability of phosphate-based formulations relative to the TBS formulation was confirmed for EO25DS after 4 weeks of storage at 40 °C, as detailed in Appendix C.2. Furthermore, the three formulations for DS EO25 were evaluated for the impact of exposure to 24 h of agitation stress at RT in two containers (polycarbonate—PC and polyethylene terephthalate glycol—PETG). Visible cloudiness was observed for the TBS-based formulation incubated in PETG bottles (Figure 5), whereas this was not seen for formulations incubated in PC bottles (a clear visual appearance was observed for all three formulations) (Figure 5).

#### 3.1.2. Selection of the Final DS and DP Formulation

Two phosphate formulations that demonstrated superior performance compared to the TBS formulation are presented in Table 2.

The sorbitol-containing phosphate formulation variant (DP P7SMC/DS P7SM25) exhibited greater robustness across all evaluated stress conditions, as per sections (Section 3.1 and Section 3.2.1 and Appendix C), resulting in its selection as an optimal formulation for ExPEC4 DP and ExPEC DS. The selected formulation, including excipient concentrations and their functions, is presented in Table 3.

#### 3.1.3. Formulation Confirmation for ExPEC Multivalent Drug Products

**Figure 6:**
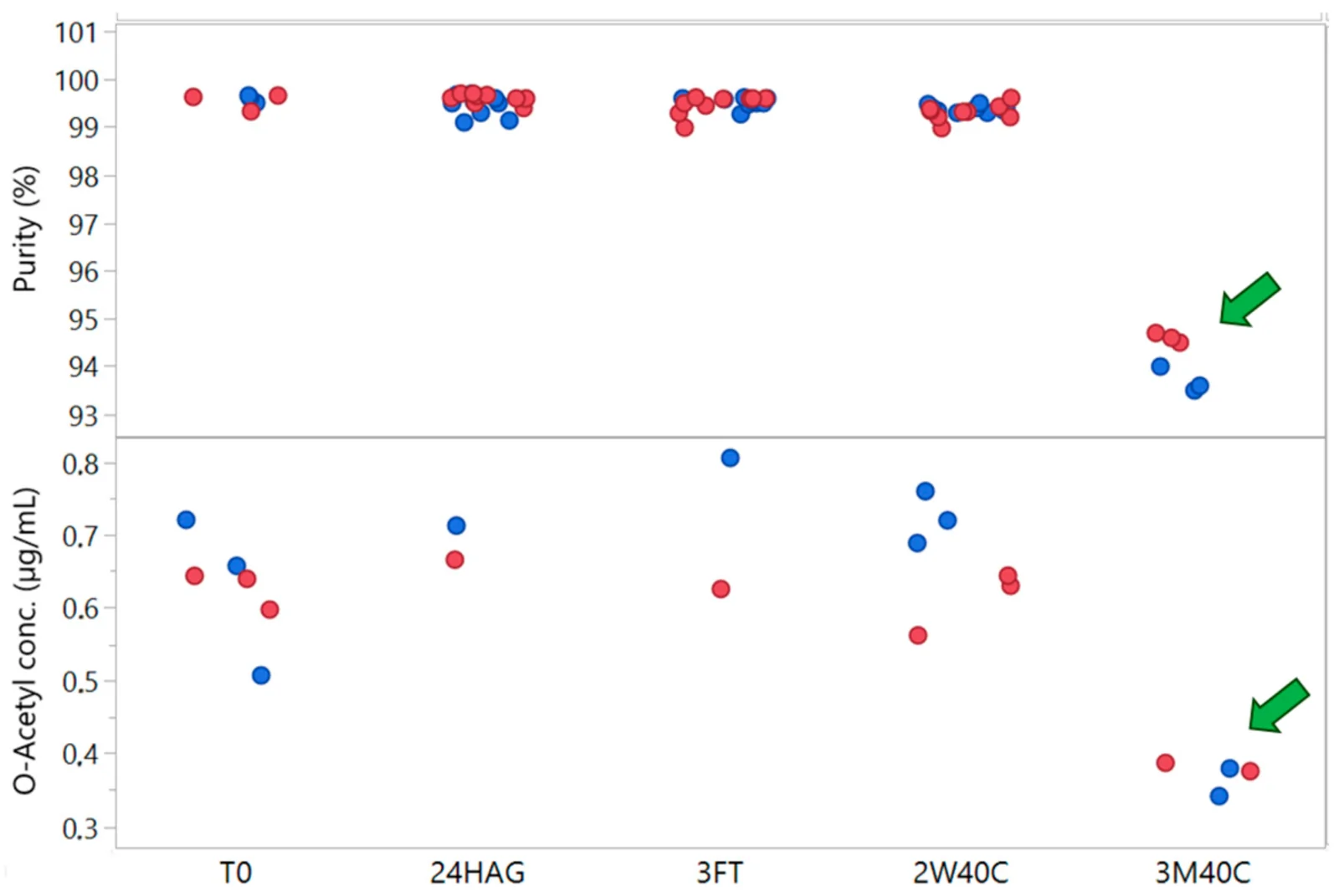
Purity and O-acetyl concentration results for ExPEC10V DP (HD—high dose, LD—low dose); 24H AG = 24 h agitation at RT; 3FT= 3 freeze/thaw cycles; 2W 40C= 2 weeks storage at 40 °C; and 3M40C, 3 months storage at 40 °C. HD—high dose (blue), LD—low Dose (red). Green arrow: attributes impacted by exposure to 40 °C for 3 months. O-acetyl concentration: *n* = 1 for 24HAG and 3FT, *n* = 2 3M40C, and *n* = 3 for T0 and 2W40C.

Due to the progressive addition of serotypes to the drug product during vaccine candidate development—from the initial ExPEC4V through ExPEC10V to the Phase-3 (9V) candidate—formulation-confirmation studies were performed to verify that the formulation developed for the 4V vaccine was suitable for other ExPEC multivalent drug products. In this way, it was possible to continuously justify the use of the platform approach applied during the clinical progression of the candidate vaccine from 4V to 9V. The outcome of the study, which evaluated the stability of the ExPEC10V DP in the selected formulation (Table 3) under storage and stress conditions described in Table A4, is presented in Figure 6. Selected attributes—purity and degree of O-acetylation (or O-acetyl concentration)—were unchanged under stress conditions, except after 3 months at 40 °C when they declined (Figure 6, green arrows), which was consistent with the stability profile of the ExPEC4V in the same formulation, thus confirming the selected formulation for the 10V DP.

### 3.2. Compatibility

#### 3.2.1. Early Compatibility—Tungsten Spiking

Tungsten spiking was performed prior to final formulation selection on three formulations selected from the formulation screening work: phosphate and TBS formulation (Appendix C.2) to evaluate preliminary compatibility with, e.g., prefilled syringes.

**Figure 7:**
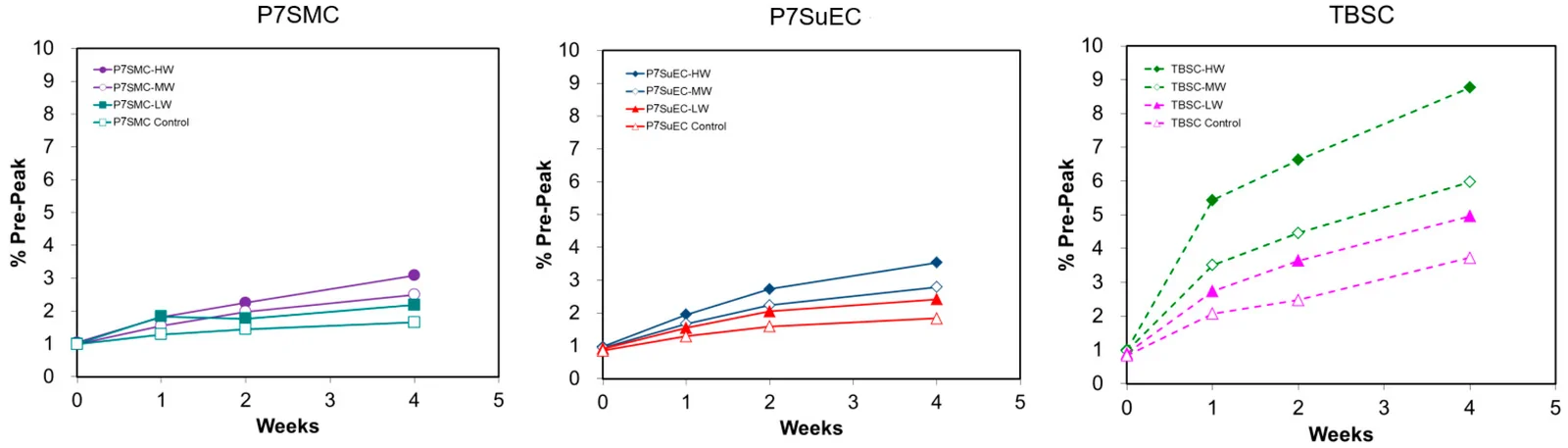
SE-HPLC Pre-Peak trends of formulations after 4 weeks at 40 °C. LW: 417 ng/mL, MW: 833 ng/mL, HW: 2083 ng/mL tungsten concentration. Designations used: Histidine (H); K/Na phosphate (P); Tris buffered saline (TBS); phosphate buffered saline (PBS); pH (in case of phosphate formulations) = 6.5 (65) 7.0 (7), 7.5 (75); sorbitol (S); sucrose (Su); EDTA (E); methionine (M); ExPEC4V DP (C); EO25 DS (25).

Formulations were spiked with various concentrations of tungsten extract after agitation stress or following 4 weeks of incubation at 40 °C. Among the assessed attributes, only purity by SE-HPLC was affected in the selected formulations, and this was observed only after storage at 40 °C for 4 weeks. There, a correlation between aggregate formation and tungsten concentration was observed, supporting the superiority of phosphate formulations relative to the TBS formulation (Figure 7, Table S9).

#### 3.2.2. Compatibility of Selected Formulation with Contact Materials

**Figure 8:**
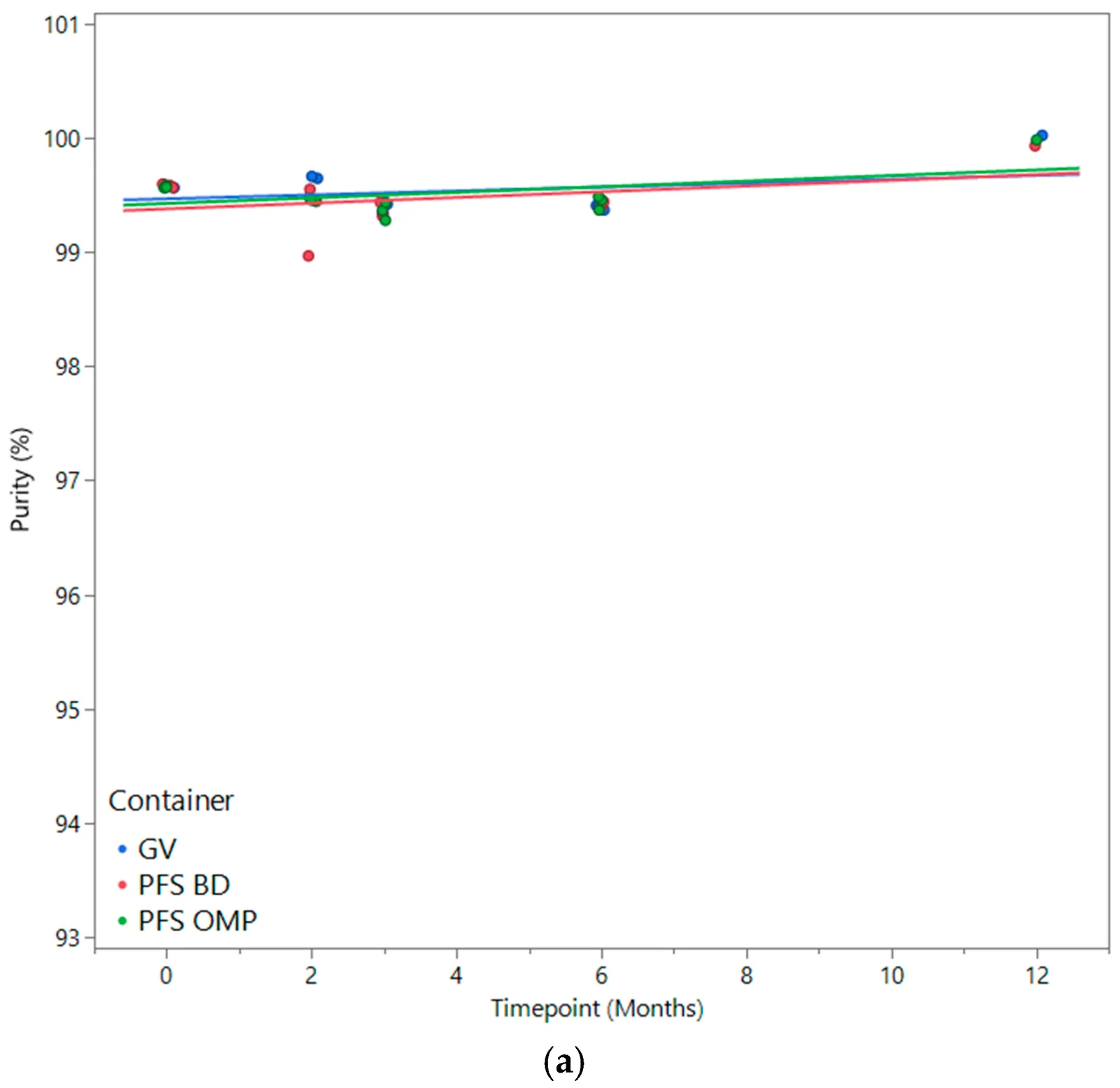

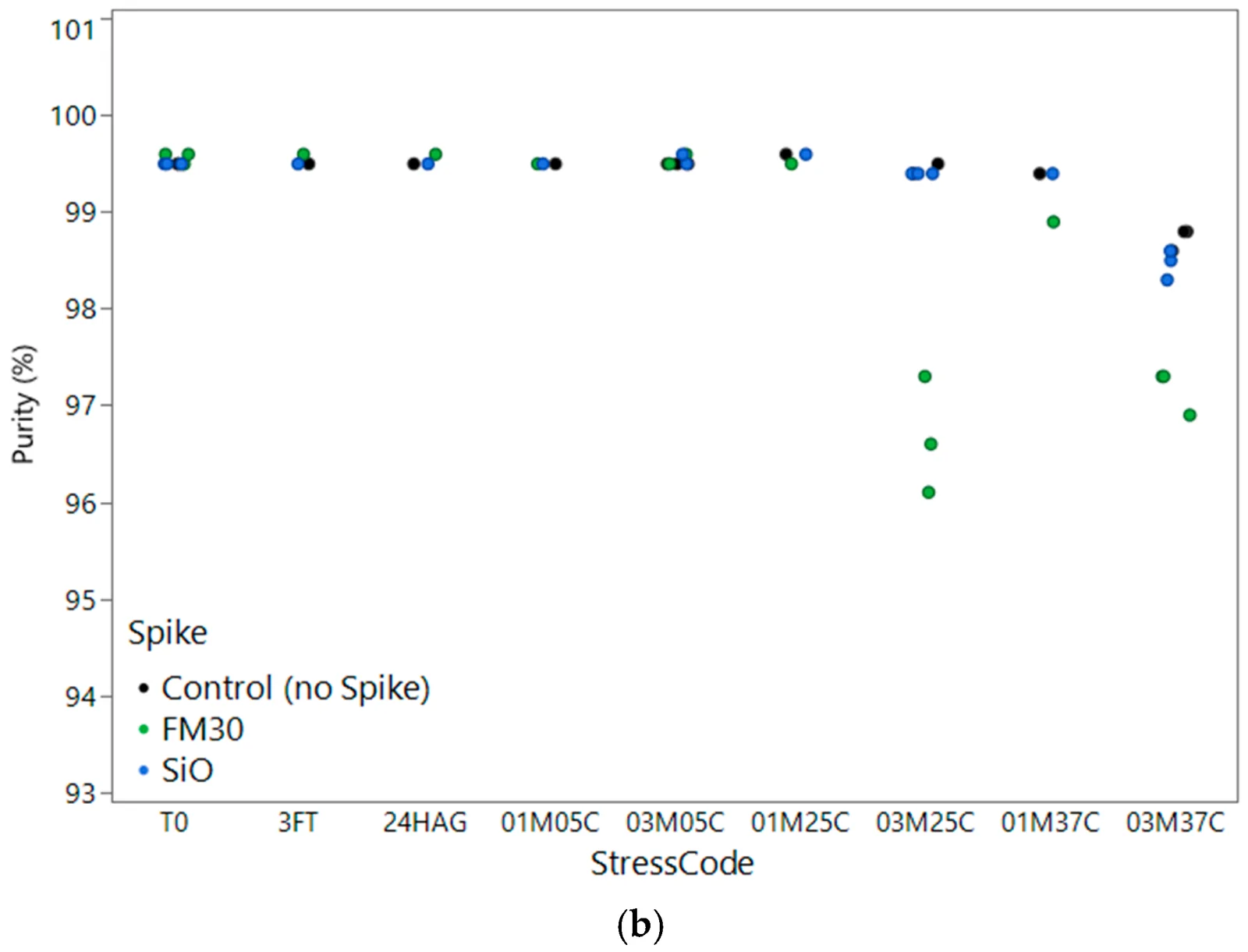
(**a**) Purity (%) by SE-HPLC of ExPEC9V DP at 5 °C up to 12 months. Depicted are the individual values, as well as the linear fit over time. (GV)- ExPEC9V DP in glass vials (PFS OPM)—ExPEC9V DP in PFS from OMPI (Nipro glass), and (PFS BD)—ExPEC9V DP in PFS from Becton, Dickinson (Fiolax glass). *n* = 3 for all timepoints. (**b**) Purity (%) by SE-HPLC of ExPEC9V DP control (black), FM30 (green) and SiO emulsion (blue) spiked. 3FT = 3 freeze/thaw cycles, 24HAG = agitation at 200 rpm for 24 h at RT, M = months. *n* = 3 for T0, 03M05C, 03M25C and 03M37C; *n* = 1 for 3FT, 24HAG, 01M05C, 01M25C, 01M37C.

After selecting ExPEC9V as the Ph3 DP, a series of studies was conducted to confirm the formulation and expand understanding of product properties. Compatibility with prefilled syringes from two suppliers (Nipro glass (OMPI) and Fiolax glass (BD)) was demonstrated in a comparative stability assessment, including a glass-vial control (ExPEC9V DP in borosilicate glass vials (GV), serving also as an additional formulation confirmation step for the ExPEC9V Ph3 vaccine candidate). Data shows stability in PFS and glass vials for at least 12 months at 2–8 °C (Figure 8a), confirming the findings from early formulation screening work. Exposure to silicone oil and FM30 cap rubber did not affect stability for assessed attributes across storage and stress conditions, except for 3 months at 25 °C and 37 °C, where a slight increase in aggregation was observed in FM30-spiked samples (Figure 8b).

The formulation is also proven to be compatible with materials in manufacturing, including linear low-density polyethylene (LLDPE), low-density polyethylene (LDPE), stainless steel, and residual peroxide amounts, as described in D2 Compatibility with materials in manufacturing.

### 3.3. Formulation Robustness

Deviations from the target DP composition that may occur during buffer preparation or in the formulation step were assessed in a formulation robustness study. The robustness of the ExPEC9V DP Ph3 candidate in vials and PFS is discussed in detail in E. Formulation robustness ExPEC9V. This study served to determine acceptable excipient ranges and their impact on DP quality and stability. Results from this study generally inform formulation buffer specification ranges, the criticality analysis, and DP manufacturing control strategy, and can support assessments of potential deviations. All formulations evaluated in this study passed the acceptance criteria for the tested potential (p)CQAs for 6 months of real-time storage at 5 °C, 7 freeze/thaw (FT) cycles, as well as 3 days of agitation at room temperature (RT), as detailed in E. Formulation robustness ExPEC9V.

### 3.4. ICH Long-Term Stability and Photostability

**Figure 9:**
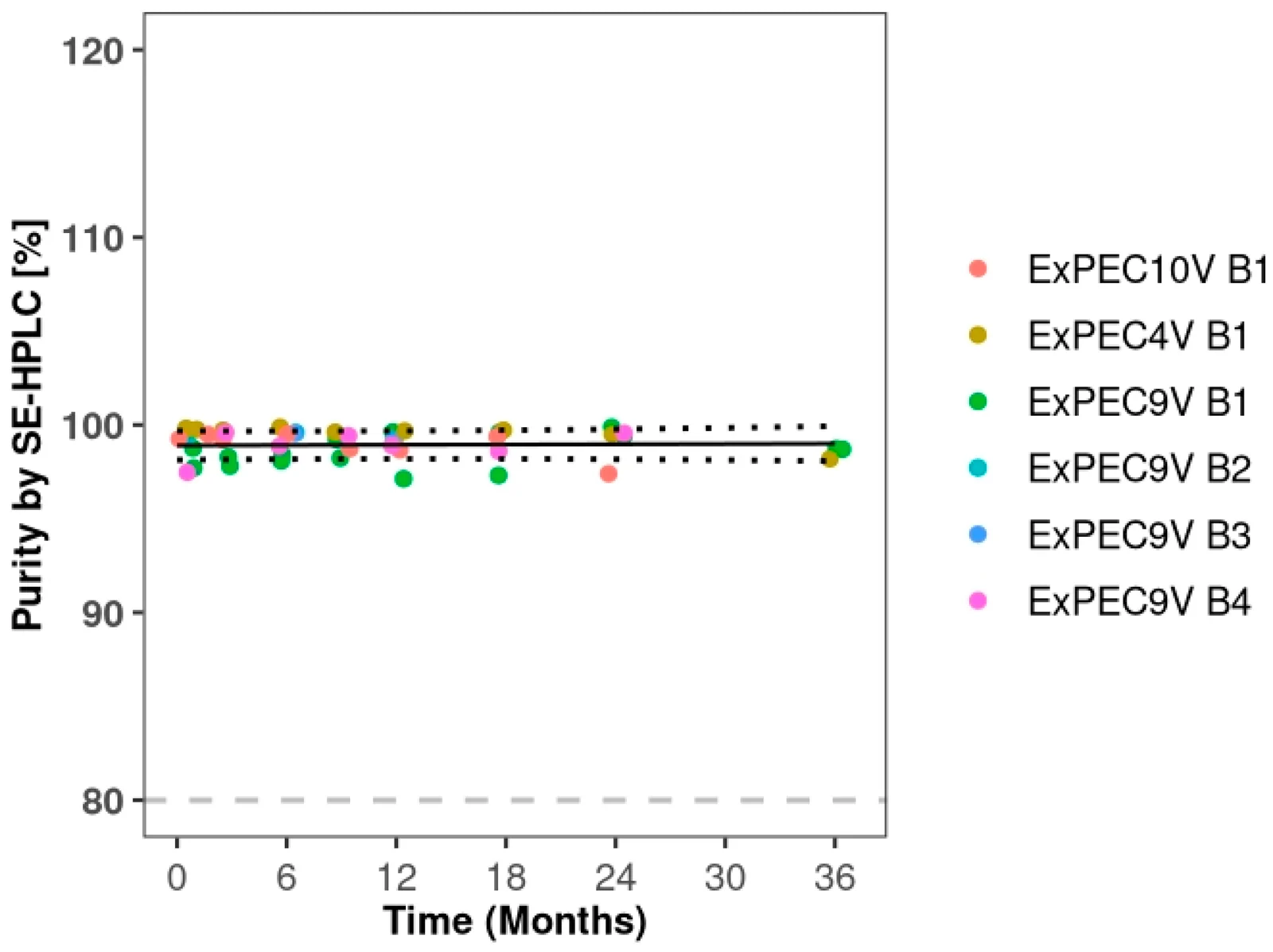
Stability data: purity by SE-HPLC from ExPEC DP batches, 36 months of storage at 2–8 °C. Grey dashed lines represent DP Ph3 specifications (Table S1). B= Batch (B1, 1 batch of ExPEC10V and ExPEC4V; B1, 2, 3, 4 = 4 batches of ExPEC9V). Stability data: ExPEC4V B1 and ExPEC9V B1 up to 36 months, ExPEC10V B1 and ExPEC9V B4 up to 24 months, and ExPEC9V B2 and ExPEC9V B3 up to 12 months.

The long-term stability of ExPEC DP and DS formulation was further confirmed in the ICH stability studies carried out with several clinical batches of ExPEC DP with different valencies, including the four batches of Ph3 9V DP (Figure 9) and multiple ExPEC DS batches (Figure S1). The stability of all the ExPEC DP batches was confirmed to be within Ph3 specifications (listed in Table S1) and no degradation was observed for up to and including 36 months at 2–8 °C, as demonstrated here by purity by SE-HPLC (Figure 9).

The stability of several ExPEC DS batches, some of which have been proven to be stable at −70 ± 10 °C for 48 months, is presented in Figure S1.

Additionally, the photostability of multivalent ExPEC was confirmed with ExPEC 10V DP, which was subjected to rigorous light exposure of 1.2 million lux-hours of visible light and 200 W-hr/m^2^ of integrated UV energy, particularly within the spectral range of 300–400 nm (ICH Q1B). The results, focusing on the total protein content, indicated that there was no significant impact on glycoconjugate structure or its physicochemical properties after light exposure (Table 4).

## 4. Conclusions

The formulation selected through screening studies performed with ExPEC 4V DP and EO25 DS was subsequently confirmed for higher valency candidates, including ExPEC 10V and the Ph3 candidate ExPEC 9V DP, as well as all evaluated DS serotypes.

The Ph3 ExPEC 9V DP exhibited sustained stability under refrigerated storage of 2–8 °C, compatibility with multiple primary containers and manufacturing contact materials, and resistance to degradation during routine handling and upon exposure to harsh light. This supports reliable production and product integrity across various conditions and light-exposure stresses. As a result, it allowed for the storage of materials for extended periods and higher flexibility during manufacturing, facilitating a substantially improved clinical supply chain, including storage, and shipment.

By adopting a platform-based formulation and connecting early formulation screening with late-stage requirements (manufacturing robustness, multi-container compatibility, and multi-year stability), our story illustrates how an early, robust formulation strategy can be successfully translated into a late-stage, scalable vaccine candidate. These integrated data, spanning early design to late-stage requirements, may offer a novel and generalizable approach for ongoing or future development programs involving multivalent bioconjugates based on dEPA carrier protein and O-polysaccharide antigens.

## 5. Patents

2WO2018077853A1—“ExPEC glycoconjugate vaccine formulations”.

## Figures and Tables

**Table 1:** Factors of the present ExPEC9V DP formulation robustness study in vials and PFS. The target/midpoint excipient concentrations and pH value are presented in bold.

	Extremes		Target	Extremes	
Excipient	Very Low	Low	Midpoint	High	Very High
Phosphate buffer (mM)	2.5	5	**10**	15	20
Sorbitol (% *w*/*w*)	1.5	3	**5**	8	10
Methionine (mM)	2.5	5	**10**	15	20
Polysorbate 80 (% *w*/*w*)	-	0.01	**0.02**	0.04	-
pH	-	6.5	**7.0**	7.5	-
Total polysaccharide (μg/mL)	-	123	**176**	229	-
**Primary containers:** Glass vial, pre-filled syringe					

**Table 2:** Two lead candidate phosphate formulations DP P7SMC/DS P7SM25 and DP P7SuEC/DS P7SuE25.

**Buffer**	Phosphate: 6.19 mM KH_2_PO_4_, 3.81 mM Na_2_HPO_4_, pH 7.0	
**Tonicity modifier**	5% sorbitol *or*	8% sucrose
**Antioxidants**	Methionine (10 mM) *or*	EDTA (1 mM)
**Surfactant**	0.02% (*w*/*w*) polysorbate 80 (PS80)	

**Table 3:** Final formulation (pH 7.0) selected for ExPEC DS and 4VDP based on the formulation screening studies.

Component	Function	Concentration
KH_2_PO_4_	Buffer	6.19 mM
Na_2_HPO_4_	Buffer	3.81 mM
Sorbitol	Cryoprotectant and tonicity agent	5% (*w*/*w*)
Methionine	Antioxidant	10 mM
Polysorbate 80	Surfactant	0.02% (*w*/*w*)

**Table 4:** Total protein concentration on light-stressed DP material.

Material	Total Protein by A280 [μg/mL]	
	Control	Light
ExPEC10V DP	320	324

**Figure A1:**
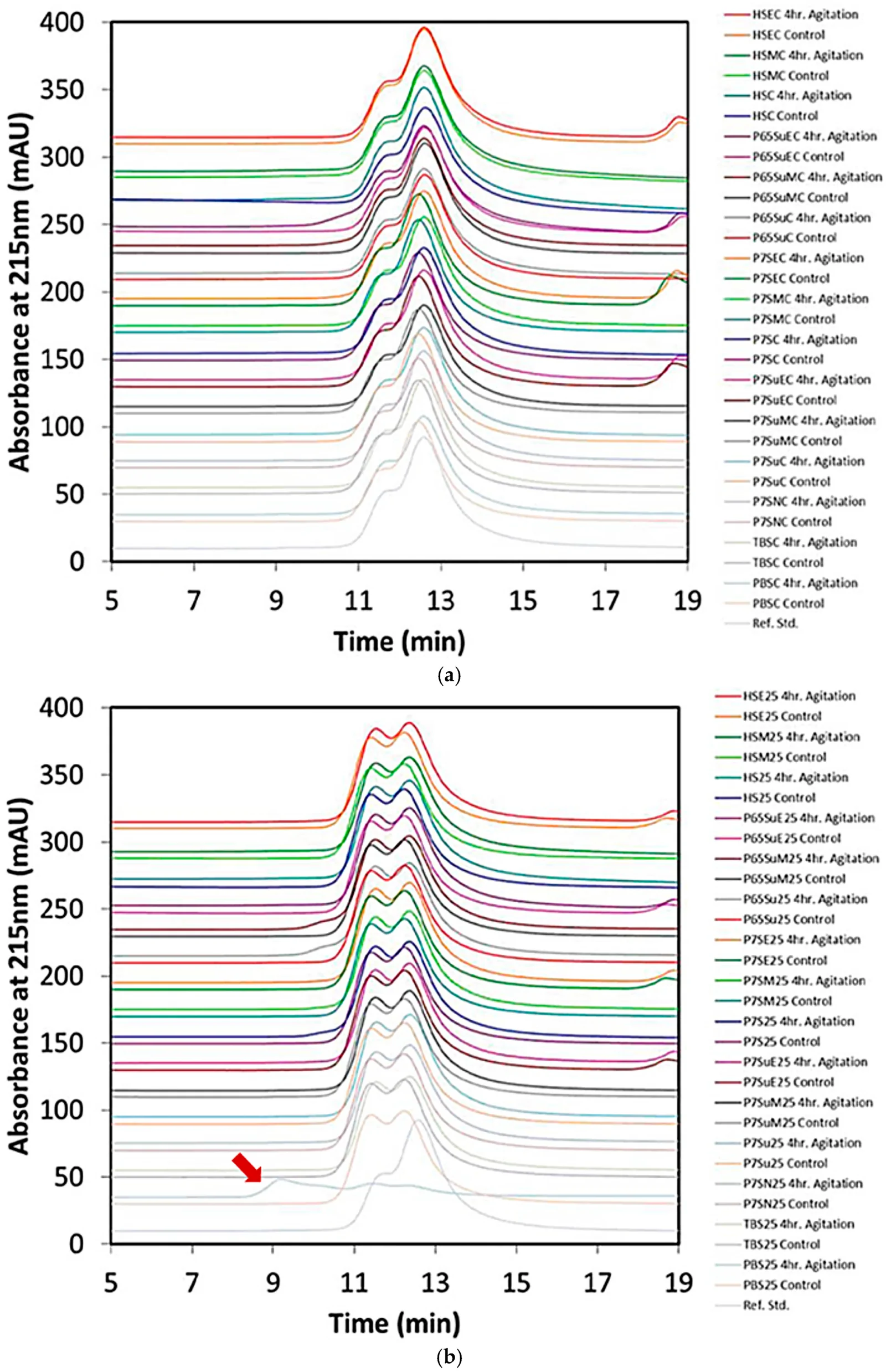
SE-HPLC results for ExPEC4V DP (**a**) and EO25 DS (**b**) formulations (agitated for 4 h at RT and static control). The formulation matrix is described in Table A2. Designations used: Histidine (H); K/Na phosphate (P); Tris buffered solution (TBS); phosphate buffered saline (PBS); pH (in the case of phosphate formulations) = 6.5 (65) 7.0 (7), 7.5 (75); sorbitol (S); sucrose (Su); EDTA (E); methionine (M); ExPEC4V DP (C); EO25 DS (25).

**Figure A2:**
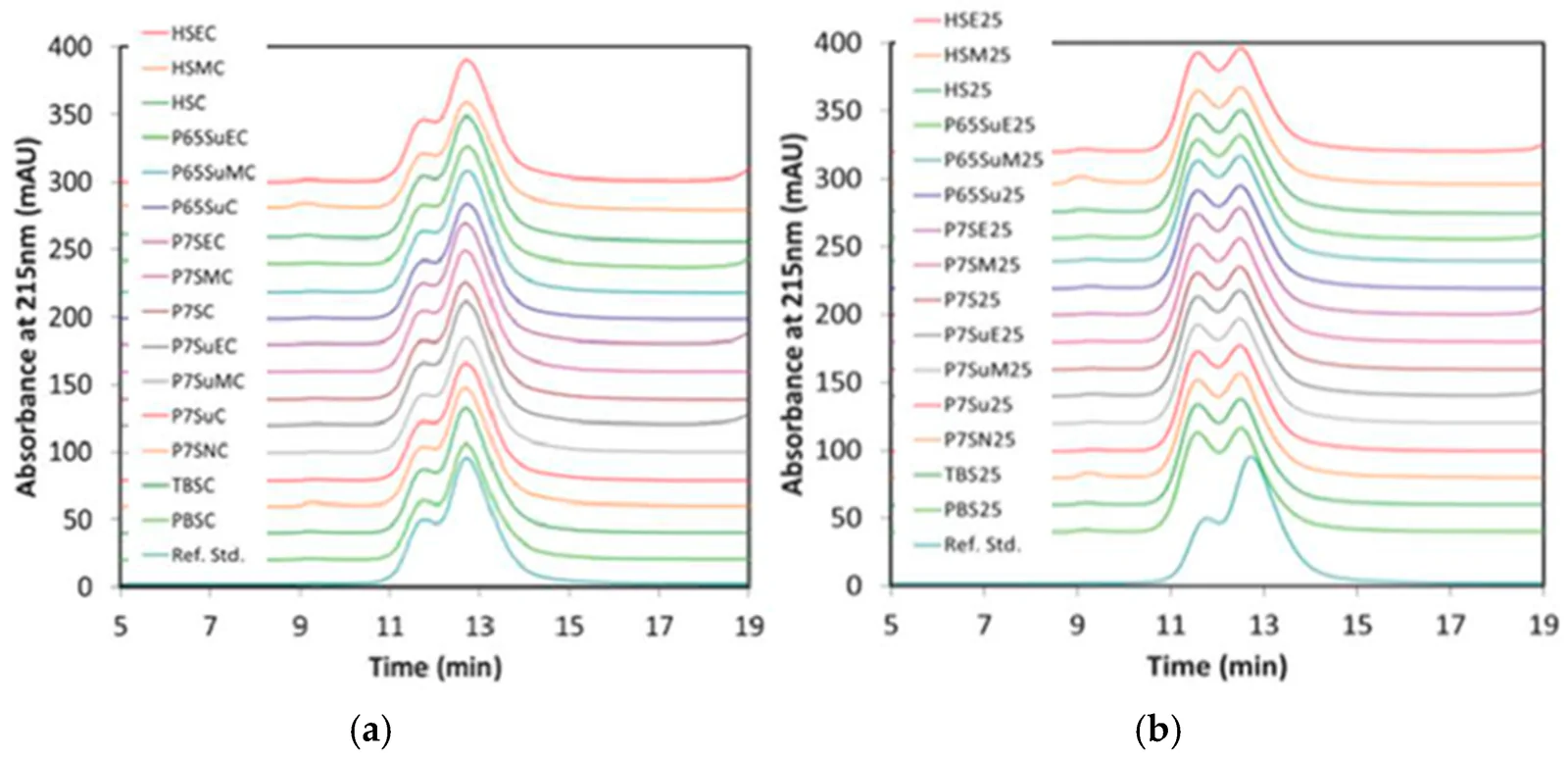
SE-HPLC chromatogram of ExPEC DP (**a**) and EO25 DS (**b**) formulations after 4 weeks at 40 °C. Formulation matrix described in Table A2. Designations used: Histidine (H); K/Na phosphate (P); Tris buffered solution (TBS); phosphate buffered saline (PBS); pH (in the case of phosphate formulations) = 6.5 (65) 7.0 (7), 7.5 (75); sorbitol (S); sucrose (Su); EDTA (E); methionine (M); ExPEC4V DP (C); EO25 DS (25).

**Figure A3:**
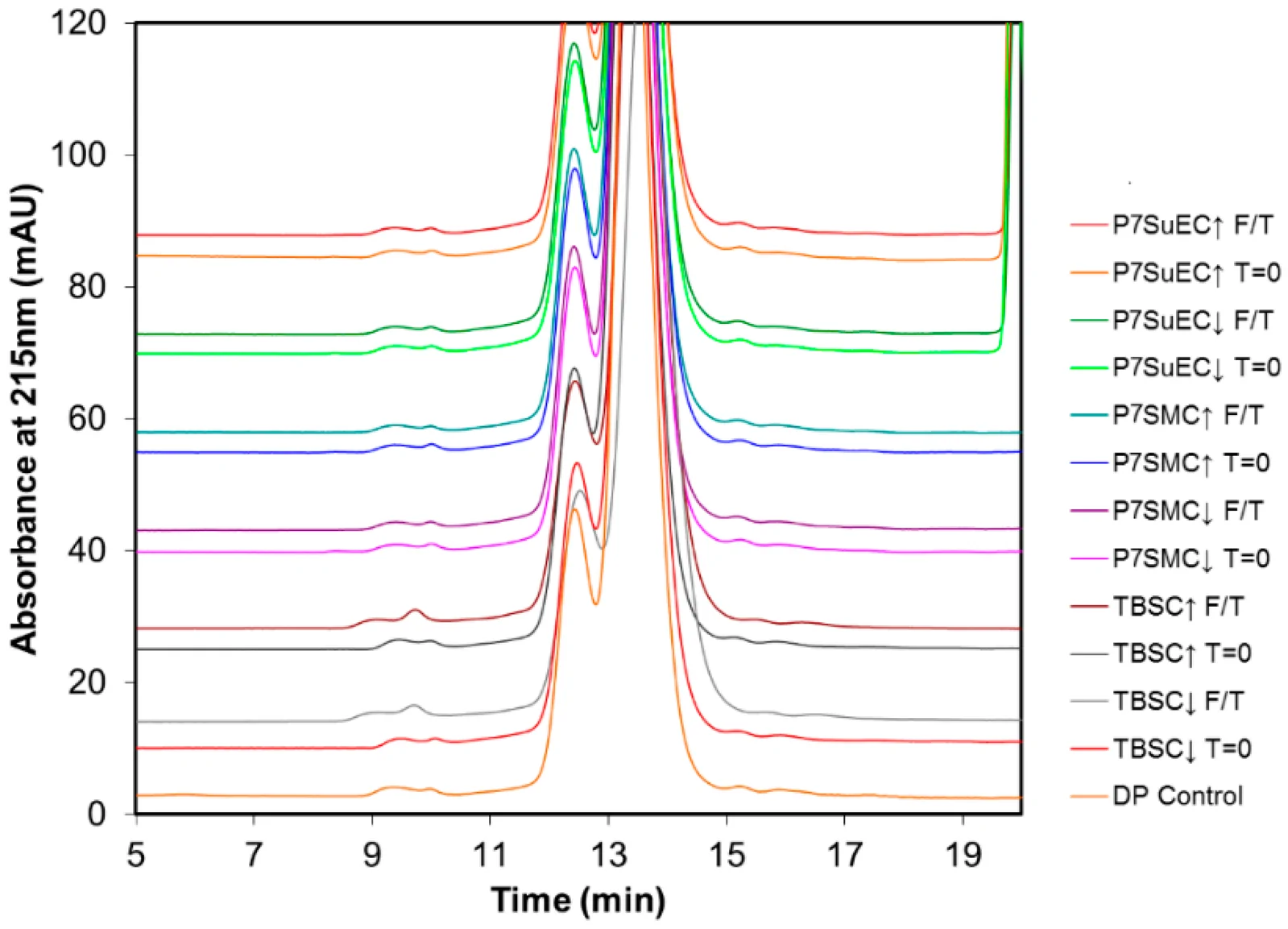
SE-HPLC chromatogram of ExPEC4V DP formulations, T = 0 and Freeze/thaw (↑: upright, ↓: inverted). Designations used: Histidine (H);K/Na phosphate (P); Tris buffered solution (TBS); phosphate buffered saline (PBS); pH (in the case of phosphate formulations) = 6.5 (65) 7.0 (7), 7.5 (75); sorbitol (S); sucrose (Su); EDTA (E); methionine (M); ExPEC4V DP (C); EO25 DS (25).

**Figure A4:**
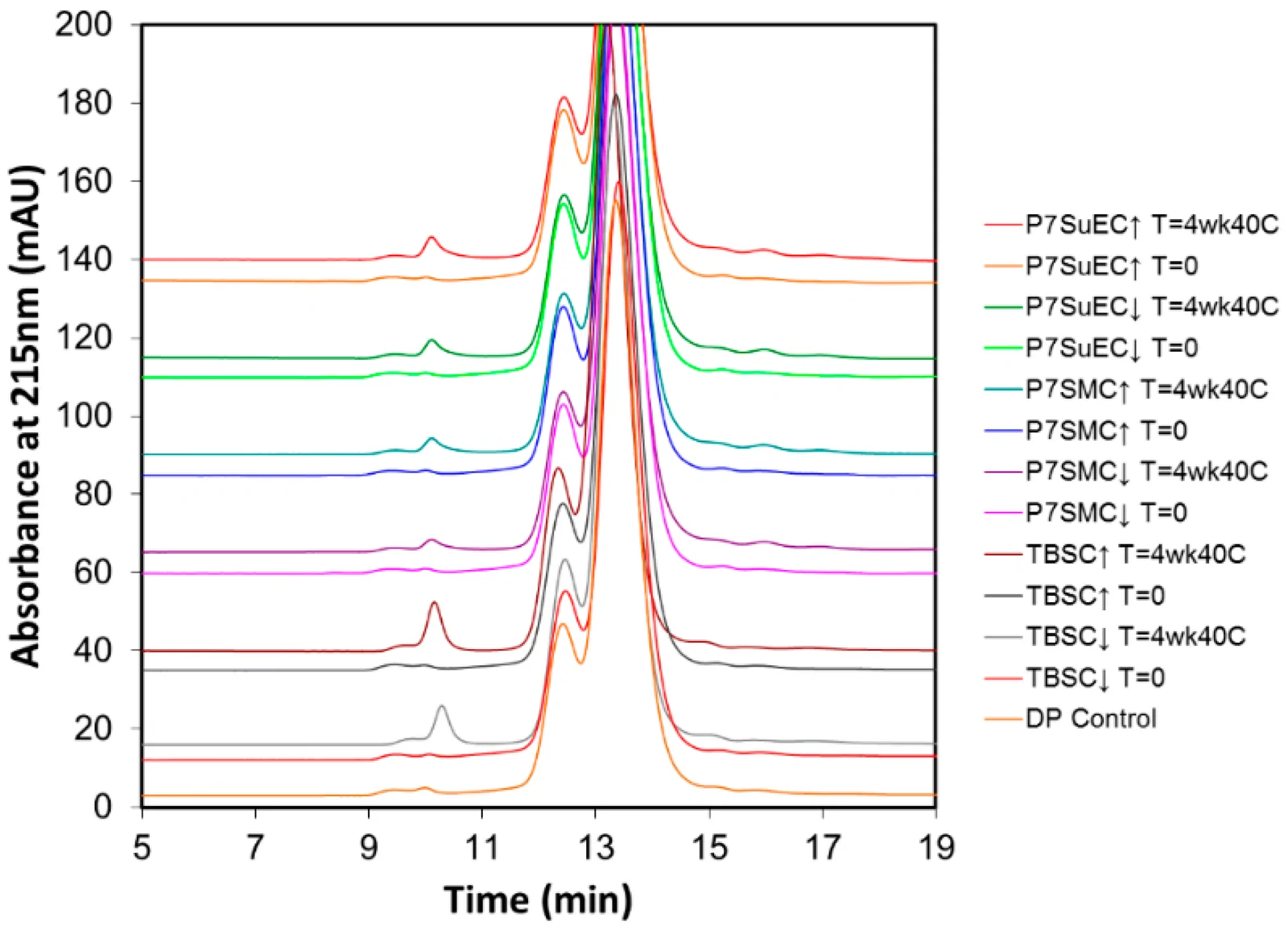
SE-HPLC peak percentages of ExPEC DS formulations after 12 weeks of storage at 40 °C. (↑: upright, ↓: inverted). Designations used: Histidine (H); K/Na phosphate (P); Tris buffered solution (TBS); phosphate buffered saline (PBS); pH (in the case of phosphate formulations) = 6.5 (65) 7.0 (7), 7.5 (75); sorbitol (S); sucrose (Su); EDTA (E); methionine (M); ExPEC4V DP (C); EO25 DS (25).

**Figure A5:**
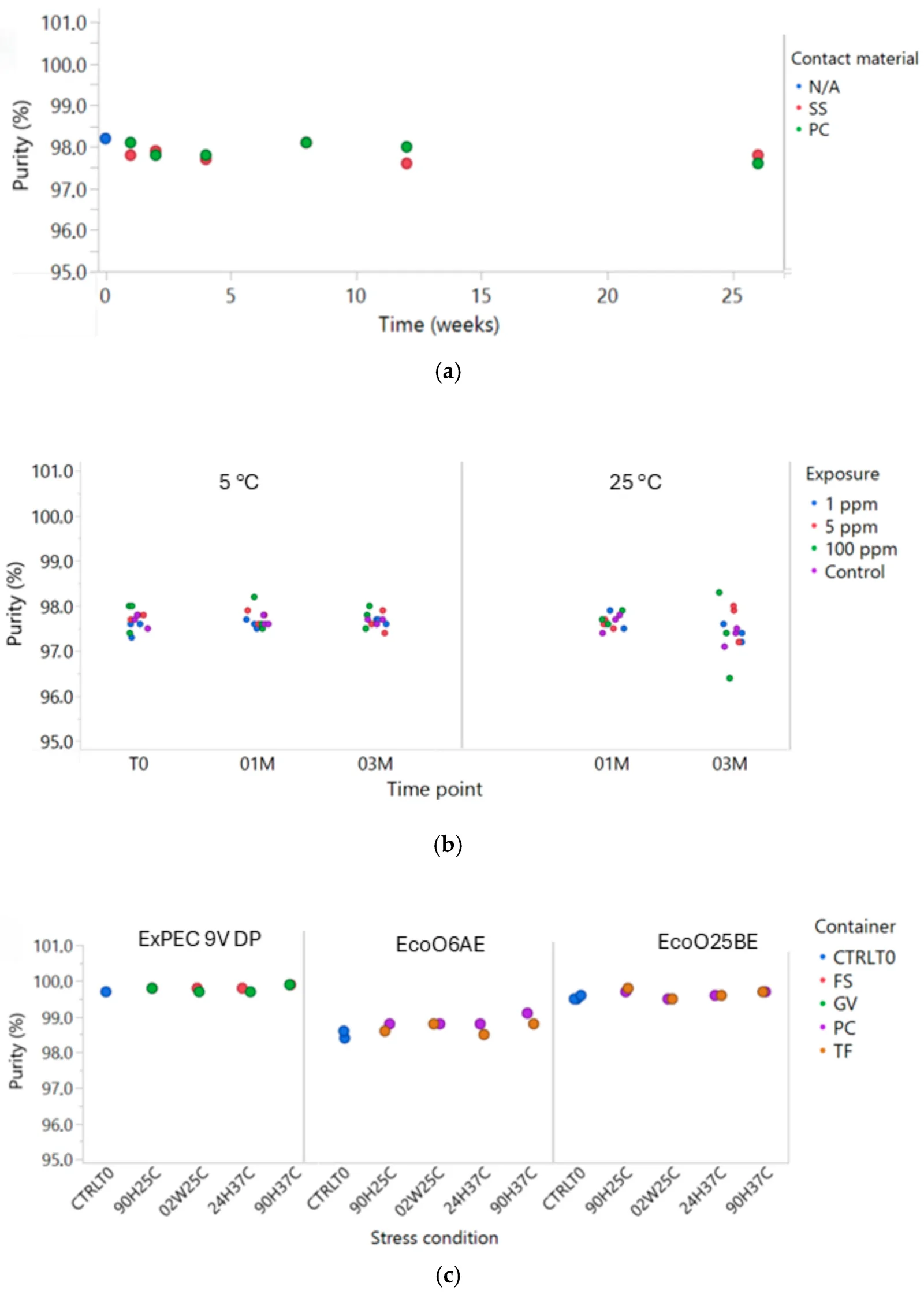
Purity as determined by SEC of (**a**): ExPEC9V DP at 5 °C for up to 26 weeks. PC: samples incubated in PC bottles; SS: Incubate in stainless steel containers. (**b**) ExPEC9V DP by SE-HPLC over time (months): blue -control—non-spiked; red—spiked with 1 ppm H_2_O_2_; green—spiked with 5 ppm H_2_O_2_; purple—spiked with 100 ppm H_2_O_2_. (**c**) ExPC9V DP, EcoO6AE DS and EcoO25BE DS after exposure to 5 different stresses in different containers. GV—Glass vial; TF-Tepoflex Biocontainer (LDPE contact material); PC—Polycarbonate; FS—FlexSafe150 mL bags (LLDPE contact material).

**Figure A6:**
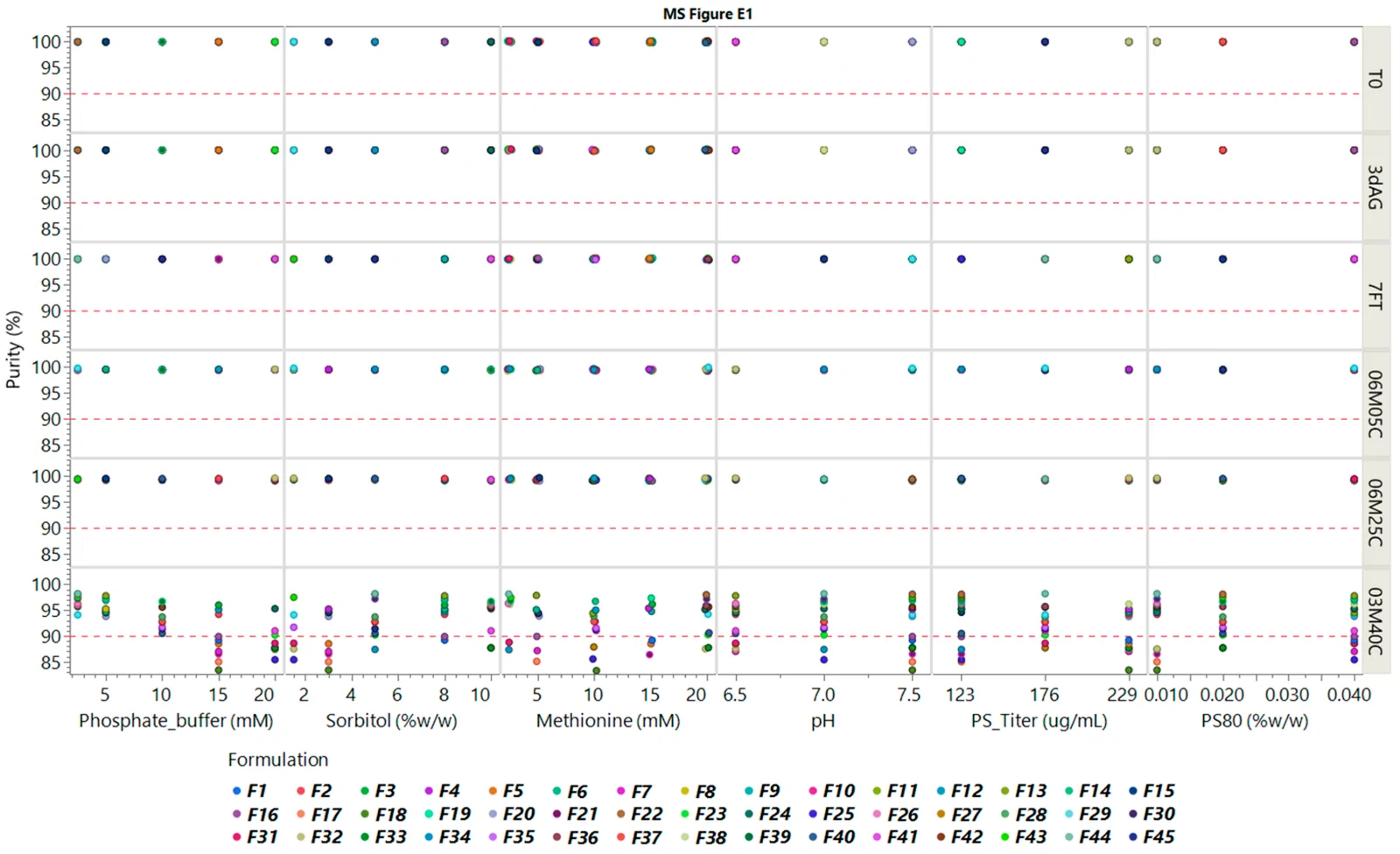
Purity (%) results (by SEC-HPLC) for the 45 tested formulations and storage/stress conditions, *n* = 1. Within each subfigure, each marker is the value of a single measurement (i.e., each subfigure contains the data for all 45 formulations). However, for each condition (“Stress code”, i.e., each row of subfigures) all subfigures contain the same measurements, but they are sorted according to the values of the excipients/titer/pH in order to visualize the impact of each of those factors of the DoE. Markers are not always visible as they often have near-identical values and therefore are stacked on top of each other. The red dashed lines indicate the acceptance criterion of ≥90% purity. Stress codes: 3dAG = 3 days of agitation at room temperature; 7FT = 7 freeze/thaw cycles; 06M05C—6 months storage at 2–8 °C; 06M25C—6 months storage at 25 °C; 03M40C—3 months storage at 40 °C.

**Figure A7:**
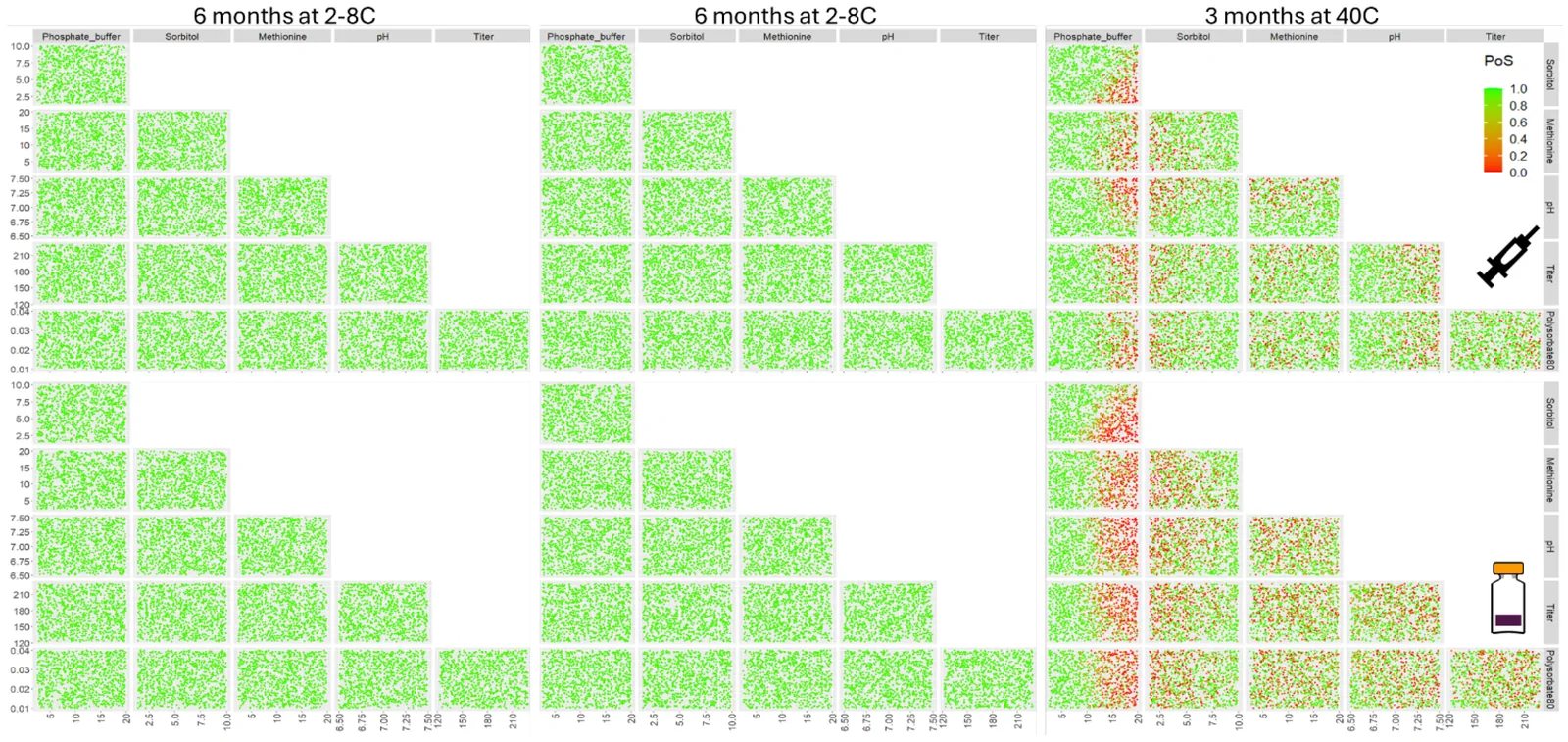
Probability of success (PoS) to meet the specification of purity ≥90%, as a function of the tested DoE parameters for product-related purity. PoS was calculated for the storage conditions of 6 months at 5 °C (**left** panels), 6 months at 25 °C (**middle** panels) and 3 months at 40 °C (**right** panels) for syringe (**top** panels) and vial (**bottom** panels) containers. For each of these 6 panels, PoS is quantified using color coding for the combinations of—from left to right—concentrations of phosphate buffer, sorbitol, methionine, pH and titer, together with (from **top** to **bottom**) sorbitol, methionine, pH, titer and PS80 concentrations.

**Figure A8:**
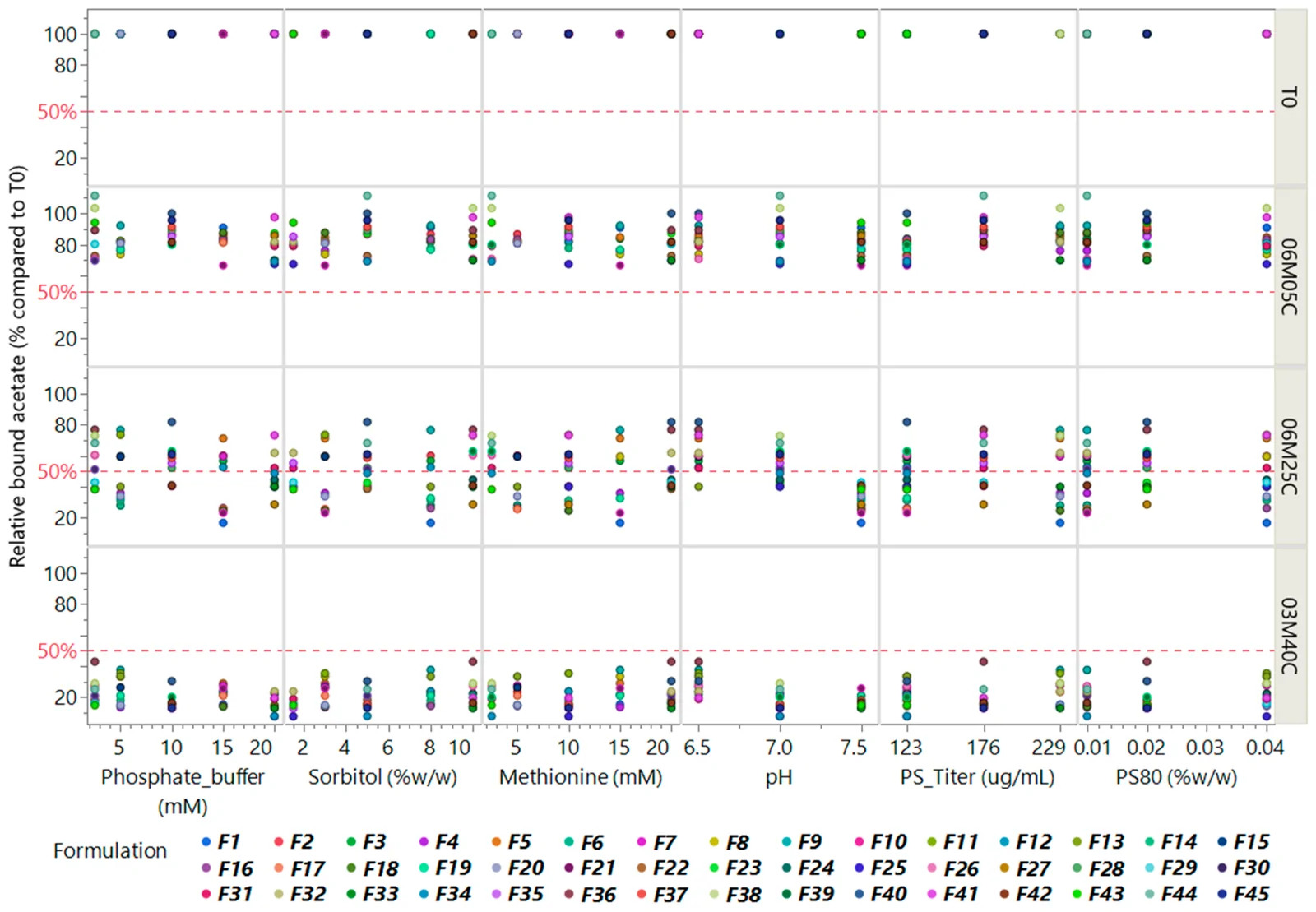
Degree of O-acetylation (% relative to T0) results (by enzymatic colorimetry) for the 45 tested formulations and storage/stress conditions, *n* = 1. Within each subfigure, each marker is the value of a single measurement (i.e., each subfigure contains the data of all 45 formulations). However, for each condition (“Stress code”, i.e., each row of subfigures) all subfigures contain the same measurements, but they are sorted according to the values of the excipients/titer/pH in order to visualize the impact of each of those factors of the DoE. Markers are not always visible as they sometimes have near-identical values and therefore are stacked on top of each other. The red dashed lines indicate the acceptance criterion of ≥50% (relative to T0). Stress codes: 06M05C—6 months storage at 2–8 °C; 06M25C—6 months storage at 25 °C; 03M40C—3 months storage at 40 °C.

**Figure A9:**
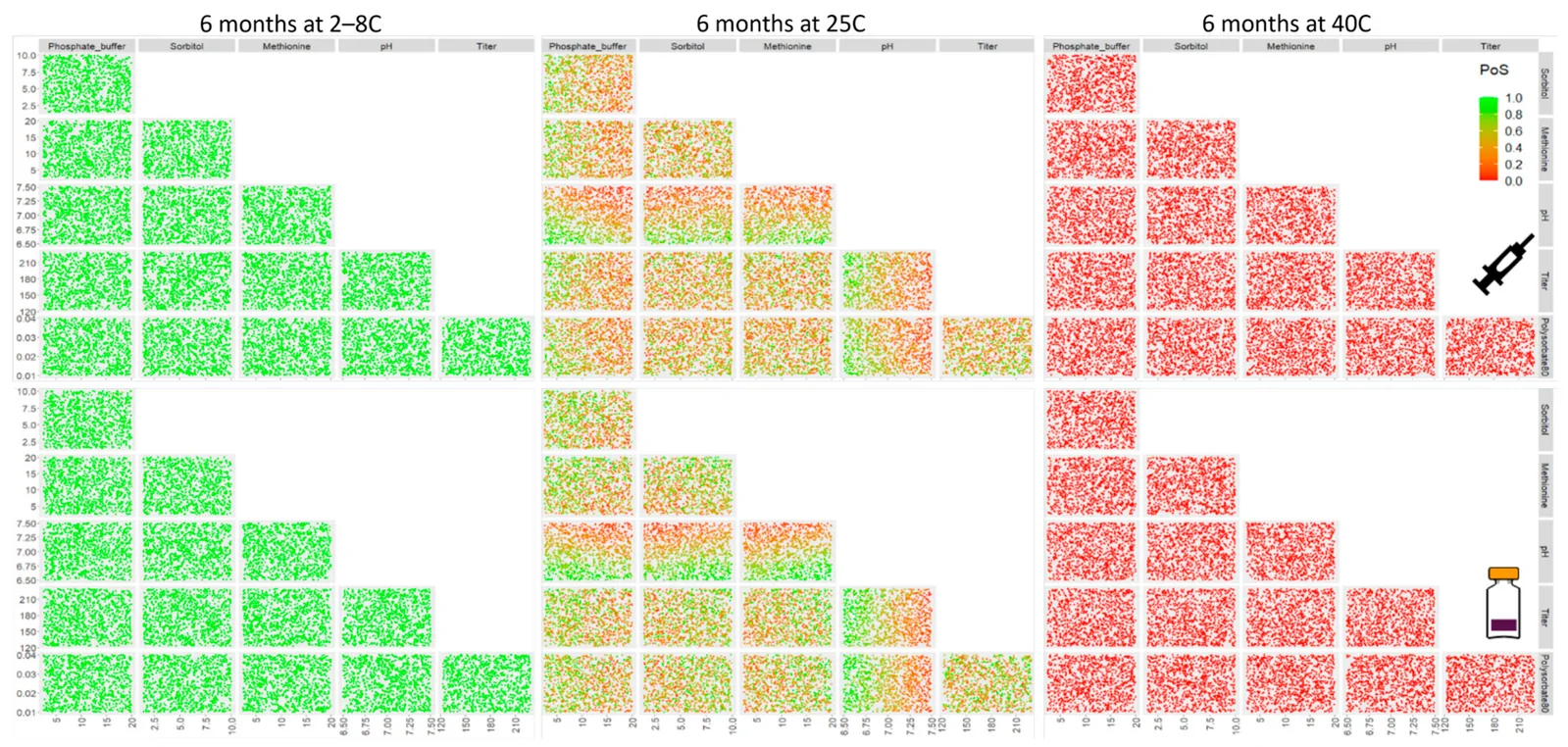
Probability of success (PoS) to meet the specification of ≥50% degree of O-acetylation relative to the T0 value, as a function of the tested DoE parameters. PoS was calculated for the storage conditions of 6 months at 2–8 °C (**left** panels), 6 months at 25 °C (**middle** panels) and 3 months at 40 °C (**right** panels) for syringe (**top** panels) and vial (**bottom** panels) containers.

## Data Availability

All data that support the findings of this study are available from the corresponding author upon request.

## Supplementary Materials

The following supporting information can be downloaded at: https://www.mdpi.com/article/10.3390/vaccines14080690/s1, Supplementary Figure S1: Stability data of ExPEC DS 10 serotypes: Purity by SE-HPLC, 36 months storage at −70 ± 10 °C. Grey dashed lines present DS Ph3 specifications. Different colors represent different DS; Supplementary Table S1: Specifications (selected attributes discussed in this manuscript) for release and stability testing of ExPEC9V drug product for Phase 3 clinical studies. The DP specifications have undergone revisions during the development progression, results measured were evaluated as per requirements applicable at the time; Supplementary Table S2: SE-HPLC peak percentages for ExPEC4V DP formulations (agitated and control). Designations used: Histidine (H); K/Na phosphate (P); Tris buffered saline (TBS); phosphate buffered saline (PBS); pH (in case of phosphate formulations) = 6.5 (65) 7.0 (7), 7.5 (75); sorbitol (S); sucrose (Su); EDTA (E); methionine (M); ExPEC4V DP (C); EO25 DS (25); Supplementary Table S3: SE-HPLC peak percentages for EO25 DS formulations (agitated and control). Designations used: Histidine (H); K/Na phosphate (P); Tris buffered saline (TBS); phosphate buffered saline (PBS); pH (in case of phosphate formulations) = 6.5 (65) 7.0 (7), 7.5 (75); sorbitol (S); sucrose (Su); EDTA (E); Methionine (M); ExPEC4V DP (C); EO25 DS (25); Supplementary Table S4: SE-HPLC peak percentages for ExPEC4V DP and ExPEC DS formulations (freeze/thaw). Designations used: Histidine (H); K/Na phosphate (P); Tris buffered saline (TBS); phosphate buffered saline (PBS); pH (in case of phosphate formulations) = 6.5 (65) 7.0 (7), 7.5 (75); sorbitol (S); sucrose (Su); EDTA (E); methionine (M); ExPEC4V DP (C); EO25 DS (25); Supplementary Table S5: SE-HPLC peak percentages of ExPEC formulations after 12 weeks at 5 °C. Designations used: Histidine (H); K/Na phosphate (P); Tris buffered saline (TBS); phosphate buffered saline (PBS); pH (in case of phosphate formulations) = 6.5 (65) 7.0 (7), 7.5 (75); sorbitol (S); sucrose (Su); EDTA (E); methionine (M); ExPEC4V DP (C); EO25 DS (25); Supplementary Table S6: SE-HPLC peak percentages of three final ExPEC4V formulations (T = O and Freeze/thaw). Designations used: Histidine (H); K/Na phosphate (P); Tris buffered saline (TBS); phosphate buffered saline (PBS); pH (in case of phosphate formulations) = 6.5 (65) 7.0 (7), 7.5 (75); sorbitol (S); sucrose (Su); EDTA (E); methionine (M); ExPEC4V DP (C); EO25 DS (25). F/T—Freeze/thaw cycle; Supplementary Table S7: SE-HPLC peak percentages of ExPEC formulations after 4 weeks at 40 °C. Designations used: Histidine (H); K/Na phosphate (P); Tris buffered saline (TBS); phosphate buffered saline (PBS); pH (in case of phosphate formulations) = 6.5 (65) 7.0 (7), 7.5 (75); sorbitol (S); sucrose (Su); EDTA (E); methionine (M); ExPEC4V DP (C); EO25 DS (25). Supplementary Table S8: SE-HPLC peak percentages of three final ExPEC4V formulations after 12 weeks of storage at 40 °C. Designations used: Histidine (H); K/Na phosphate (P); Tris buffered saline (TBS); phosphate buffered saline (PBS); pH (in case of phosphate formulations) = 6.5 (65) 7.0 (7), 7.5 (75); sorbitol (S); sucrose (Su); EDTA (E); methionine (M); ExPEC4V DP (C); EO25 DS (25); Supplementary Table S9: SE-HPLC peak areas of tungsten compatibility formulations after 4 weeks at 40 °C. Designations used: Histidine (H); K/Na phosphate (P); Tris buffered saline (TBS); phosphate buffered saline (PBS); pH (in case of phosphate formulations) = 6.5 (65) 7.0 (7), 7.5 (75); sorbitol (S); sucrose (Su); EDTA (E); methionine (M;; ExPEC4V DP (C); EO25 DS (25).

## Abbreviations

AD	Analytical Development
AT	Ambient Temperature
AG	Agitation
A280	Absorbance at 280 nm
AMR	Antimicrobial Resistance
API	Active Pharmaceutical Ingredient
BD	Becton Dickinson
BLA	Biologics License Application
CRM197	Cross-reactive material 197
CMC	Chemistry, Manufacturing and Controls
CQAs	Critical Quality Attributes
DT	Diphtheria Toxoid
dEPA	Detoxified Exotoxin
d	Day
DLS	Dynamic Light Scattering
DoE	Design of Experiments
DP	Drug Product
DS	Drug Substance
EDTA	Ethylenediaminetetraacetic Acid
EPA	Exotoxin A
EMA	European Medicines Agency
EPAR	European Public Assessment Report
ExPEC	Extraintestinal pathogenic *Escherichia coli*
ExPEC4V/ExPEC9V/ExPEC10V	4-, 9-, or 10-valent ExPEC vaccine candidate
FDA	Food and Drug Administration
FT	Freeze/thaw
h	Hour
GV	Glass vial
HD/LD/MD	High dose/Low dose/Medium dose
HPLC	High-performance liquid chromatography
ICH	International Council for Harmonisation
ICH Q1B	International Council for Harmonisation guideline Q1B on photostability testing
IC-PAD	Ion chromatography with pulsed amperometric detection
IED	Invasive ExPEC disease
IEF	Isoelectric focusing
LDPE	Low-density polyethylene
LDAO	Lauryl dimethylamine oxide
LLDPE	Linear low-density polyethylene
MALS	Multi-angle light scattering
m	Month
MFI	Micro-flow imaging
MW	Molecular weight
OMPI	Ompi (glass primary packaging supplier)
PBS	Phosphate-buffered saline
PETG	Polyethylene terephthalate glycol
PC	Polycarbonate
pCQA	Potential critical quality attribute
PFS	Pre-filled syringe
Ph3	Phase 3
PoS	Probability of success
PRP	Polyribosyl–ribitol–phosphate
PS	Polysaccharide
PS80	Polysorbate 80
QbD	Quality by design
rEPA	Recombinant exotoxin A
RH	Relative humidity
RP-HPLC	Reversed-phase high-performance liquid chromatography
RT	Room temperature
SEC/SE-HPLC	Size-exclusion (high-performance) liquid chromatography
SoloVPE	Variable pathlength extension spectroscopy
SS	Stainless steel
TBS	Tris-buffered saline
TT	Tetanus toxoid
USP	United States Pharmacopeia
UV	Ultraviolet
w	Week
WHO	World Health Organization
WES	Wes™ capillary-based Western system

## Appendix A

### Appendix A.1. ExPEC Drug Substance and Drug Products—Composition

The polysaccharide concentration of ExEPC EO25 DS was 200 µg/mL. The serotype composition and concentrations of ExPEC vaccines with different valences presented in this manuscript are described in Table A1. The total protein content varied from an average of 220 µg/mL for the 4V ExPEC vaccine candidate to an average of 700 µg/mL in the 9V ExPEC vaccine candidate and an average of 500 µg/mL in EO25 DS, preserving the PS/protein ratio of 0.2–0.4 (this reflects the average PS chain length and the degree of glycosylation, which both define the MW distribution of the active ingredients).

**Table A1:** Serotype composition and concentrations of ExPEC vaccine with different valencies.

DP Name	Serotype (µg/mL)									
	O1A	O2	O4	O6A	O8	O15	O16	O18A	O25B	O75
ExPEC 4V	8	8	N/A	8	N/A	N/A	N/A	N/A	16	N/A
ExPEC 10V (LD) ^1^	8	8	8	8	8	8	8	8	16	8
ExPEC 10V (HD) ^2^	16	16	16	16	16	16	16	16	32	16
ExPEC 10V (MD) ^3^	16	8	8	16	8	8	8	8	32	8
ExPEC9V	16	16	16	16	N/A	16	16	16	32	32

^1^—LD—Low Dose; ^2^—HD—High Dose; ^3^—MD—Medium Dose.

### Appendix A.2. Bioconjugation Process

Conjugation of glycan antigens to a carrier protein in vaccine formulations enabled enhanced long-term immunological memory and increased vaccine stability [44,45,46]. The technique of in vivo bioconjugation removed limitations of the original conjugation technology, including challenges of combining multiple individually conjugated serotypes in a single vial and purifying the O- antigen as (delipidated) polysaccharide [44,46,47]. Bioconjugation utilizes engineered *E. coli* cells, which express the O-polysaccharide of the different O-serotypes in the *E. coli* periplasm, in the presence of oligosaccharyl transferase (PglB), and the carrier protein [48]. This process allows for the extraction and purification of a fully assembled glycoconjugate vaccine. As a result of the in vivo glycoconjugate vaccine manufacturing process, the active substances produced are of relatively high purity, contrasting with the typical high levels of heterogeneity that arise from chemical cross-linking.

## Appendix B. Materials and Methods Detailed Tables

**Table A2:** ExPEC4V DP and EO25 DS formulation matrix for the formulation screening studies. Formulations 1–15: ExPEC4V DP; Formulations 16–30 EO25 DS. Bolded formulations were selected and further assessed for stability and compatibility, including tungsten compatibility. Three selected EO25 DS formulations were also assessed for compatibility with primary packaging (Figure 5). Exc.—Excipient; Meth.—Methionine; So—Sorbitol; Su—Sucrose; Surf.—Surfactant (PS80).

Form.	Form Code	Buffer	pH	Exc. 1	Exc. 2	Exc. 3	Surf. PS80 Conc.
1	HSEC (DP)	15 mM Histidine	6.5	5% So	1 mM EDTA	-	0.02%
2	HSMC (DP)	15 mM Histidine	6.5	5% So	10 mM Meth.	-	0.02%
3	HSC (DP)	15 mM Histidine	6.5	5% So	-	-	0.02%
4	P65SuEC (DP)	8.37 mM KH_2_PO_4_/1.63 mM Na_2_HPO_4_	6.5	8% Su	1 mM EDTA	-	0.02%
5	P65SuMC (DP)	8.37 mM KH_2_PO_4_/1.63 mM Na_2_HPO_4_	6.5	8% Su	10 mM Meth.	-	0.02%
6	P65SuC (DP)	8.37 mM KH_2_PO_4_/1.63 mM Na_2_HPO_4_	6.5	8% Su	-	-	0.02%
7	P7SEC (DP)	6.19 mM KH_2_PO_4_/3.81 mM Na_2_HPO_4_	7.0	5% So	1 mM EDTA	-	0.02%
8	P7SMC (DP)	6.19 mM KH_2_PO_4_/3.81 mM Na_2_HPO_4_	7.0	5% So	10 mM Meth.	-	0.02%
9	P7SC (DP)	6.19 mM KH_2_PO4/3.81 mM Na_2_HPO_4_	7.0	5% So	-	-	0.02%
10	P7SuEC (DP)	6.19 mM KH_2_PO_4_/3.81 mM Na_2_HPO_4_	7.0	8% Su	1 mM EDTA	-	0.02%
11	P7SuMC (DP)	6.19 mM KH_2_PO_4_/3.81 mM Na_2_HPO_4_	7.0	8% Su	10 mM Meth.	-	0.02%
12	P7SuC (DP)	6.19 mM KH_2_PO_4_/3.81 mM Na_2_HPO_4_	7.0	8% Su	-	-	0.02%
13	P7SNC (DP)	6.19 mM KH_2_PO_4_/3.81 mM Na_2_HPO_4_	7.0	2.5% So	-	75 mM NaCl	0.02%
14	TBSC (DP)	TBS, 25 mM Tris	7.5	-	-	137 mM NaCl, 2.7 mM KCl	n/a
15	PBSC (DP)	10 mM Sodium Phosphate	6.5	-	-	150 mM NaCl	n/a
16	HSE25 (DS)	15 mM Histidine	6.5	5% So	1 mM EDTA	-	0.02%
17	HSM25 (DS)	15 mM Histidine	6.5	5% So	10 mM Meth.	-	0.02%
18	HS25 (DS)	15 mM Histidine	6.5	5% So	-	-	0.02%
19	P65SuE25 (DS)	8.37 mM KH_2_PO_4_/1.63 mM Na_2_HPO_4_	6.5	8% Su	1 mM EDTA	-	0.02%
20	P65SuM25 (DS)	8.37 mM KH_2_PO_4_/1.63 mM Na_2_HPO_4_	6.5	8% Su	10 mM Meth.	-	0.02%
21	P65Su25 (DS)	8.37 mM KH_2_PO_4_/1.63 mM Na_2_HPO_4_	6.5	8% Su	-	-	0.02%
22	P7SE25 (DS)	6.19 mM KH_2_PO_4_/3.81 mM Na_2_HPO_4_	7.0	5% So	1 mM EDTA	-	0.02%
23	P7SM25 (DS)	6.19 mM KH_2_PO_4_/3.81 mM Na_2_HPO_4_	7.0	5% So	10 mM Meth.	-	0.02%
24	P7S25 (DS)	6.19 mM KH_2_PO_4_/3.81 mM Na_2_HPO_4_	7.0	5% So	-	-	0.02%
25	P7SuE25 (DS)	6.19 mM KH_2_PO_4_/3.81 mM Na_2_HPO_4_	7.0	8% Su	1 mM EDTA	-	0.02%
26	P7SuM25 (DS)	6.19 mM KH_2_PO_4_/3.81 mM Na_2_HPO_4_	7.0	8% Su	10 mM Meth	-	0.02%
27	P7Su25 (DS)	6.19 mM KH_2_PO_4_/3.81 mM Na_2_HPO_4_	7.0	8% Su	-	-	0.02%
28	P7SN25 (DS)	6.19 mM KH_2_PO_4_/3.81 mM Na_2_HPO_4_	7.0	2.5% So	-	75 mM NaCl	0.02%
29	TBS25 (DS)	TBS	7.5	-	-	-	n/a
30	PBS25 (DS)	10 mM Sodium Phosphate	6.5	-	-	150 mM NaCl	n/a

**Table A3:** DS and DP samples and contact materials assessed in the early and late development studies. Studies: 1—Formulation screening and packaging compatibility (tungsten spiking); 2—Formulation confirmation; 3—Developmental stability in vials & PFS; 4—Spiking with silicone oil and FM30 rubber material compatibility with PFS; 5—Formulation robustness; 6—Compatibility with LDPE, LLDPE; 7—Stainless steel compatibility; 8—Peroxide compatibility; 9—ICH stability; 10—Photostability.

Study	DS and DP Tested	Contact Materials
1	EO25 DS ExPEC4V DP	2R Type 1 borosilicate glass vials, Schott (Mainz, Germany) 13 mm 4432/50 chlorobutyl stopper with Flurotec coating (West, Exton, PA, USA) Tungsten concentration: 417 ng/mL, 833 ng/mL, 2083 ng/mL (Tungsten Extract 106,000 ng/mL (in water) Becton Dickinson, BD, Franklin Lakes, NJ, USA
	EO2 DS EO25 DS	PC bottle 20 mL (Thermo Scientific, Waltham, MA, USA) PETG bottle 30 mL (Thermo Scientific, Waltham, MA, USA)
2	ExPEC10V DP (high dose HD, low dose LD)	2R Type 1 borosilicate glass vials, Schott (Mainz, Germany) 13 mm 4432/50 chlorobutyl stopper with Flurotec coating (West, Exton, PA, USA)
3	ExPEC9V DP	1.0 mL short Nipro glass PFS with 4432/50 chlorobutyl stopper with Flurotec coating and FM30 tip cap, BD, Franklin Lakes, NJ, USA 1.0 mL short Fiolax glass PFS with 4432/50 chlorobutyl stopper with Flurotec coating and FM30 tip cap, OMPI, Piombino Dese, Italy 2R Type 1 borosilicate glass vials, OMPI, Piombino Dese, Italy 13 mm 4432/50 chlorobutyl stopper with Flurotec coating (West, Exton, PA, USA)
4	EcoO25BE DS ExPEC9V DP Formulation buffer	DOW Corning 366 dimethicone (silicone oil, DOW Corning, Midland, MI, USA) FM30 PFS tip cap (Datwyler, Altdorf, Switzerland)
5	ExPEC9V DP	1.0 mL short Fiolax glass PFS with 4432/50 chlorobutyl stopper with Flurotec coating and FM30 tip cap, OMPI 2R Type 1 borosilicate glass vials, OMPI, Piombino Dese, Italy 13mm 4432/50 chlorobutyl stopper with Flurotec coating (West, Exton, PA, USA)
6	EcoO6 DS, EcoO25 DS ExPEC9V DP	low-density polyethylene (LDPE) 50 mL bag (Meissner TepoFlex^®^ bags, Camarillo, CA, USA) with DS PC containers 5 mL (Parker, Cleveland, OH, USA) as negative control for DS linear low-density polyethylene (LLDPE) 150 mL bag (Sartorius Flexsafe^®^ bags, Göttingen, Germany) with DP Glass vials (2R Type 1 borosilicate glass vials, OMPI, Piombino Dese, Italy 13 mm 4432/50 chlorobutyl stopper with Flurotec coating (West, Exton, PA, USA) as Negative control for DP
7	ExPEC9V DP	316 L stainless-steel container 250 mL (Eagle Stainless; Warminster, PA, USA) PC bottle 20 mL (Thermo Fisher, Waltham, MA, USA) as negative control
8	ExPEC9V DP	Hydrogen peroxide: 1 ppm, 5 ppm or 100 ppm
9	ExPEC9V DP	2R Type 1 borosilicate glass vials, OMPI, Piombino Dese, Italy 13 mm 4110/40 bromobutyl stopper, with Flurotec coating (West, Exton, PA, USA)
	ExPEC9V DP ^1^	2R Type 1 borosilicate glass vials, OMPI, Piombino Dese, Italy 13 mm 4432/50 chlorobutyl stopper with Flurotec coating (West, Exton, PA, USA)
10	EcoO2E DS, EcoO6AE DS, EcoO25BE DS ExPEC 10V LD DP	NA

^1^ ExPEC9V DP batch manufactured at a different site than the other ExPEC9V DP batches.

**Table A4:** Testing conditions for the studies during the formulation development trajectory. 1—Formulation screening; 2—Early (DS) packaging compatibility; 3—Formulation robustness; 4—Formulation confirmation; 5—Stability in vials & PFS; 6—Spiking with silicone oil and FM30 rubber material (PFS compatibility); 7—Photostability; 8—Compatibility with LDPE, LLDPE; 9—Stainless steel compatibility; 10—Peroxide compatibility; 11—ICH stability. AT—Ambient temperature; RT—Room temperature; h—hour; d—day; w—week; m—month; in.—inverted; up.—upright; acc.—accelerated.

Study	Stress Conditions			Stability Storage Conditions
	Freeze Thawing (FT)	Agitation	Other	
**1**	5 cycles −70 °C to AT	1000 rpm, 4 h, AT	Photosensitivity (ICH Q 1B option 2)	5 °C: 12 w 25 °C: 8 w 40 °C: 8 w
**2**	1 and 3 cycles, −60 °C to AT	200 rpm, 24 h, AT	200 W/m2 UV-A light: ~6 h + 1200 klux-hr visible light: ~40 h	25 °C: 7 d
**3**	7 cycles, 25 to −25 °C	200 rpm, 3 d, RT	NA	5 °C: 6 m 25 °C: 6 m 40 °C: 4 m
**4**	3 cycles, 20 °C to −60 °C	200 rpm, 24 h	NA	40 °C: 3 m
**5**	3 cycles, RT to −20 °C	200 rpm, 24 h, RT	NA	5 °C: 36 m 25 °C: 6 m 37 °C: 6 m
**6**	3 cycles, 25 °C to −25 °C	200 rpm, 24 h, RT	NA	5 °C: 1 m, 3 m 25 °C: 1 m, 3 m 37 °C: 1 m, 3 m
**7**	NA		1200 klux-hr visible light: 6 h, 200 W-hr/m2 integrated UV energy (spectral range of 300–400 nm): 10 h	NA
**8**	NA	NA	NA	25 °C: 28 d 37 °C: 90 h
**9**	NA	NA	NA	5 °C: 26 w 25 °C: 1 w
**10**	NA	NA	NA	5 °C: 48 h, 1 w, 1 m, 3 m 25 °C: 48 h, 1 w, 1 m, 3 m
**11**	NA	NA	NA	Real time (5 ± 3 °C) in. and up.: 36 m Acc. (25 ± 2 °C/60% RH ± 5%RH): 6 m Stressed (40 ± 2 °C/75%RH ± 5%RH): 3 m

**Table A5:** Attributes evaluated in supporting studies and the analytical methods employed to assess these attributes. Studies: 1—Formulation screening and early compatibility study; 2—DS compatibility with primary packaging; 3—Formulation confirmation study; 4—Stability in vials & PFS; 5—Spiking with silicone oil and FM30 rubber material (compatibility with PFS); 6—Compatibility with LDPE and LLDPE; 7—Stainless steel compatibility; 8—Peroxide compatibility; 9—Formulation robustness.

		Study/Assessment								
Attribute	Analytical Method	1	2	3	4	5	6	7	8	9
Appearance and Clarity	Visual Inspection	✓	✓	✓						
Purity, Aggregate, Cleavages	SE-HPLC	✓	✓		✓	✓	✓	✓		✓
Purity	RP-HPLC	✓	✓		✓	✓				
Protein content	SoloVPE				✓	✓	✓	✓		✓
Total protein	A280	✓		✓						
Protein integrity and purity	µSEC			✓						
Total PS content	IC-PAD			✓	✓	✓		✓		✓
Purity, Chemical Modifications	IEF	✓	✓	✓						
O-acetylation (degree of O-acetylation)	Enzymatic-colorimetric assay		✓			✓	✓	✓	✓	✓
O-acetylation	IC-CD			✓						
O-antigen glycoconjugates	WES			✓	✓	✓				
Sub-visible particles	DLS	✓	✓	✓						
Subvisible particles	MFI				✓	✓				
pH	pH	✓		✓	✓	✓				✓
Osmolality	Osmolality	✓		✓					✓	✓ ^1^
Polysorbate 80 content	UHPLC-CAD							✓		✓
Sorbitol content										✓
Methionine content	RP-HPLC								✓	✓
Density and viscosity	Density and viscosity				✓	✓				
Endotoxin	LAL			✓						

^1^. Only measured at T0.

## Appendix C. Formulation Screening Work—Detailed Results

### Appendix C.1. Initial Screening of Thirty Formulations—Impact of Agitation and Temperature Stress

Agitation stress has caused a significant impact on purity of phosphate buffered saline formulation DS PBS25 (Figure A1b, red arrow), which is seen as increases in pre-peak (+34.2%) and post-peak (+28.2%) areas (Table S3). This could have been due to a higher salt concentration in combination with a low pH. As the experimental isoelectric point (pI) of the ExPEC serotypes, per internal measurements, is ~5.5 [49], pH values that are closer to the pI could reduce the net charge, and adding NaCl could remove the remaining repulsion between conjugates. This combination of pH values close to the pI of the drug product, increased salt concentration, and the fact that no surfactant was used in these formulations are the factors that could have impacted the observed low stability and aggregate formation.

Other formulations, including histidine DP HSEC, DP HSC, DS HSM25, DS HS25, exhibited negligible impact of agitation stress, demonstrated as slight increases (+0.1% to +1.1%) in pre-peak areas. EO25 DS and ExPEC4V DP phosphate formulations at pH 7.0 were not at all susceptible to acute shear stress-induced aggregation (Figure A1a, Table S2; an illustration of a sample chromatogram showing the integration for ExPEC4V is presented in Figure 3a).

Among the conditions evaluated, storage at 40 °C for 4 weeks was the only condition that differentiated the formulations, and this was detected only by purity by SE-HPLC. After storage at 40 °C for 4 weeks, a high level of aggregate species in the histidine formulation containing sorbitol and methionine as well as phosphate formulations containing salt and sorbitol at pH 6.5 and 7.0, could be seen per SE-HPLC (Figure A2, Table S7).

### Appendix C.2. Three Final Formulations—Impact of Agitation and Temperature Stress (Including Freeze/Thaw)

Across the stress conditions assessed for the three final formulations, and across all evaluated attributes, discriminatory effects were observed after exposure to freeze/thaw cycles and after storage at 40 °C for 12 weeks.

When exposed to five consecutive freeze/thaw cycles, TBS formulations displayed slight increases in pre-peak species (+0.4 to +0.7%) when compared to their respective controls (Figure A3, Table S6) whereas no noticeable change was seen in the SE-HPLC chromatogram of the phosphate formulations irrespective of vial orientation.

ExPEC DP and DS phosphate and TBS formulations exhibited comparable behavior when stored at 5 °C and 25 °C, while a visibly higher aggregation was seen for TBS formulations when exposed to 40 °C for 12 weeks (Figure A4, Table S8).

The TBS formulations also showed a significantly higher count of particles with different sizes, especially in the inverted vials, relative to the counts in the phosphate formulations (Table A6).

**Table A6:** The MFI analysis results of ExPEC4V formulations after 5 freeze/thaw cycles.

Particle Size	P7SuEC Upright	P7SuEC Inverted	P7SMC Upright	P7SMC Inverted	TBSC Upright	TBSC Inverted
>5 μm	46	23	32	80	473	1264
>10 μm	26	3	6	17	112	381
>25 μm	6	3	0	6	3	43

Designations used: K/Na phosphate (P); Tris buffered solution (TBS); phosphate buffered saline (PBS); pH (in the case of phosphate formulations) = 6.5 (65) 7.0 (7), 7.5 (75); sorbitol (S); sucrose (Su); EDTA (E); methionine (M); ExPEC4V DP (C).

## Appendix D. Compatibility with Materials in Manufacturing—Detailed Results

The final selected Ph3 formulation was thoroughly assessed for manufacturability. The evaluation included its compatibility with different materials used in manufacturing, such as formulation vessels, as well as the residual amounts of peroxide (utilized for cleaning systems after production runs and may remain in residual amounts in the pipes). A list of contact materials evaluated is presented in Table A3.

No impact of exposure to contact materials including, LLDPE, LDPE, and stainless steel as well as residual peroxide amounts, on ExPEC stability was seen under the assessed conditions described in Table A4 for any of the assessed attributes. A detailed list of the attributes assessed and methods used in presented in Table A5, illustrated here by purity as determined using SE-HPLC (Figure A5c, Figure A5a and Figure A5b respectively).

## Appendix E. Formulation Robustness ExPEC9V—Statistical Analysis

The robustness of the 9-valent ExPEC (ExPEC9V) DP was evaluated in syringes and vials, as described in the ExPEC9V Formulation robustness study, DoE. This DoE was created using the D-optimality criterion and 42 unique formulations were generated using JMP Statistical Discovery software (versions 18). An additional three formulations were added at the center points of the formulation design space to ensure a total of two formulations with center point values were filled in vials and two in PFS. Having these four center point formulations in the DoE allowed for the assessment of the lack of fit as well as the within-formulation error of the design space model.

Stress and stability conditions associated with this study are presented and attributes and analytical methods used to analyze them are presented in Table A4 and Table A5.

Multiple linear models were fitted to the observed degradation between control conditions and stress conditions to compute the probability of meeting the acceptance criteria for all the combinations of input material (formulation factors), as advised by ICH Q8, in order to define the design space for the formulation. The probability of meeting the specifications was analyzed marginally (each degradation process analyzed separately) to identify the critical quality attributes leading to significant degradation.

Data for purity and degree of O-acetylation for all the evaluated testing conditions and formulations are presented in Figure A6 and Figure A8. PoS plots for the real-time storage condition of 25 °C for 6 months, accelerated storage condition of 25 °C for 6 months and the stressed storage condition of 40 °C for 3 months are presented in Figure A7 and Figure A9.

Formulation robustness for ExPEC 9V DP was confirmed for both syringes and vials. Specifically, regardless of primary packaging, all 45 tested formulations passed the preset study acceptance criteria for the tested attributes (purity ≥ 90%, measured by SEC-HPLC; degree of O-acetylation ≥50% relative to T_0_) for the following testing conditions: 6 months of real-time (inverted) storage at 5 °C, 7 freeze/thaw (FT) cycles as well as 3 days of agitation at room temperature (RT). Moreover, the probability of success (PoS) for meeting these preset acceptance criteria after 6 months of real-time (inverted) storage at 5 °C was ≥99% for both purity and degree of O-acetylation, regardless of primary packaging.

Thus, for these storage conditions, the design space (i.e., excipient and pH ranges listed in Table 1) of the formulation does not need to be restricted from a product quality aspect in either primary packaging.

However, not all formulations passed the acceptance criteria for the accelerated storage condition of 25 °C for 6 months, nor for the stressed storage condition of 40 °C for 3 months. Nevertheless, ExPEC9V DP is not intended to be exposed to storage at either 25 °C or 40 °C. Therefore, data from these temperature storage conditions can be used to gain additional product understanding that could potentially be used for assessing deviations during manufacturing and/or the supply chain.
