# Supplementary material for: Formulation Development of a Multivalent Bioconjugate ExPEC Vaccine Candidate: Linking Early Design to Late-Stage Stability and Manufacturability
Source: Vaccines (Basel). 2026 Aug 11;14(8):690. doi: 10.3390/vaccines14080690 (PMC13517102; doi:10.3390/vaccines14080690)
Supplement: Supplementary file 1 [file vaccines-14-00690-s001.zip › vaccines-4435949-supplementary.pdf]

vaccines

Formulation development of a multivalent bioconjugate ExPEC vaccine candidate: linking early design to late-stage stability and manufacturability: Supplementary Data

Supplementary Figure S1. Stability data of ExPEC DS 10 serotypes: Purity by SE-HPLC, 36 months storage at -70 °C±10 °C. Grey dashed lines present DS Ph3 specifications. Different colors present different DS.

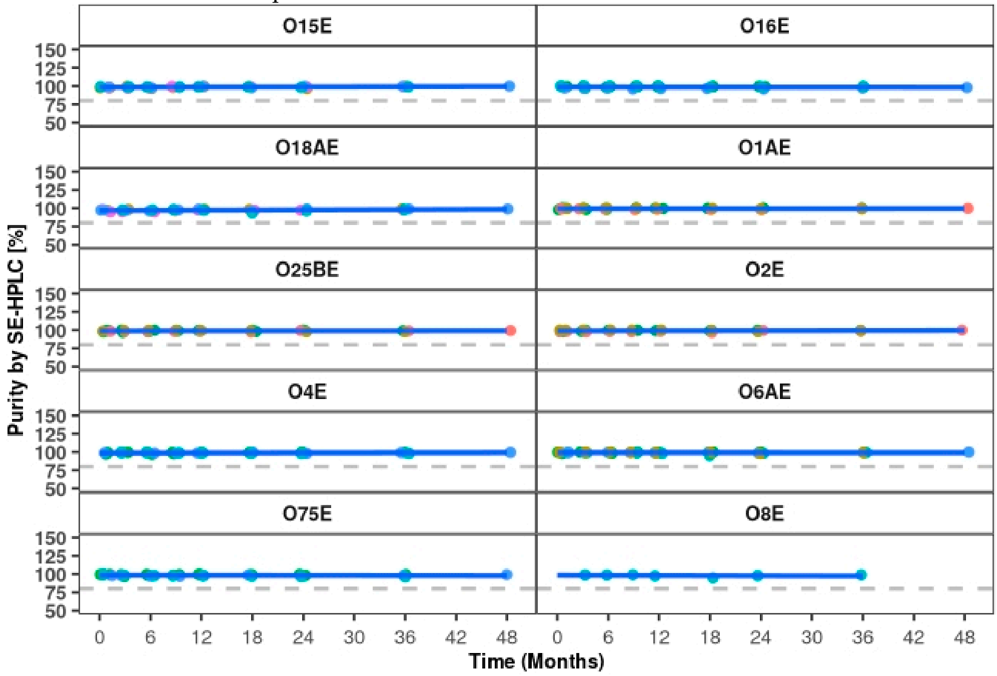

Supplementary Table S1. Specifications (selected attributes discussed in this manuscript) for Release and Stability Testing of ExPEC9V Drug Product for Phase 3 Clinical Studies. The DP specifications have undergone revisions during the development progression, results measured were evaluated as per requirements applicable at the time

Attribute	Test Method	Acceptance Criteria	
		**Release**	**Stability (Shelf Life)**
**Purity and Impurities**			
Purity	SE-HPLC	≥90%	≥90%
**Quantity**			
Total PS content	IC-PAD	123-229 μg/mL	≥ 123 μg/mL
Protein content	A_{280}	350-765 μg/mL	≥ 350 μg/mL
**General**			
pH	Ph. Eur., USP	6.7-7.3	6.7-7.3
Sub-visible particles	USP, Ph. Eur.	≥ 10 mm: ≤ 6000 particles per vial<br>≥ 25 mm: ≤ 600 particles per vial	≥10 mm: ≤ 6000 particles per vial<br>≥ 25 mm: ≤ 600 particles per vial

Supplementary Table S2. SE-HPLC peak percentages for ExPEC4V DP formulations (agitated and control). Designations used: Histidine (H); K/Na Phosphate (P); Tris Buffered Saline (TBS); Phosphate Buffered Saline (PBS); pH (in case of phosphate formulations) = 6.5 (65) 7.0 (7), 7.5 (75); Sorbitol (S); Sucrose (Su); EDTA (E), Methionine (M); ExPEC4V DP (C); EO25 DS (25)

Formulation	Pre-peak 1 (%)	Main Peak 1 (%)	Main Peak 2 (%)	Post-peak (%)	Total Area (mAU′s)
Ref. Std.	0.3	17.8	81.9	0.0	9051.3
HSEC 4hr. Agitation	0.1	17.0	82.9	0.0	8734.4
HSEC Agitation Control	0.0	17.9	82.1	0.0	9063.6
HSMC 4hr. Agitation	0.9	17.5	81.6	0.0	9493.1
HSMC Agitation Control	0.9	17.9	81.2	0.0	9427.8
HSC 4hr. Agitation	1.7	18.6	79.7	0.0	10721.4
HSC Agitation Control	0.6	16.8	82.7	0.0	8121.7
P65SuEC 4hr. Agitation	0.4	24.0	75.2	0.0	9563.3
P65SuEC Agitation Control	0.2	17.1	82.7	0.0	8654.5
P65SuMC 4hr. Agitation	0.1	17.9	82.0	0.0	8932.9
P65SuMC Agitation Control	0.1	17.1	82.8	0.0	8917.4
P65SuC 4hr. Agitation	0.3	17.2	82.5	0.0	8636.5
P65SuC Agitation Control	0.2	17.5	82.3	0.0	8545.1
P7SEC 4hr. Agitation	0.1	17.1	82.8	0.0	8685.6
P7SEC Agitation Control	0.2	17.8	82.0	0.0	8745.3
P7SMC 4hr. Agitation	0.2	17.2	82.7	0.0	8855.5
P7SMC Agitation Control	0.1	17.8	82.1	0.0	8911.8
P7SC 4hr. Agitation	0.2	17.5	82.4	0.0	8665.1
P7SC Agitation Control	0.2	17.5	82.3	0.0	8615.7
P7SuEC 4hr. Agitation	0.1	17.8	82.1	0.0	8787.9
P7SuEC Agitation Control	0.1	17.4	82.6	0.0	8636.2
P7SuMC 4hr. Agitation	0.1	17.4	82.4	0.0	8209.2
P7SuMC Agitation Control	0.1	17.8	82.1	0.0	8224.5
P7SuC 4hr. Agitation	0.3	17.9	81.8	0.0	8814.0
P7SuC Agitation Control	0.1	17.7	82.2	0.0	8613.5
P7SNC 4hr. Agitation	0.2	16.8	83.0	0.0	8897.6
P7SNC Agitation Control	0.1	17.7	82.2	0.0	8702.5
TBSC 4hr. Agitation	0.2	17.8	82.0	0.0	8794.2
TBSC Agitation Control	0.1	17.9	82.0	0.0	9050.7
PBSC 4hr. Agitation	0.2	18.9	80.8	0.0	8086.6
PBSC Agitation Control	0.0	17.7	82.2	0.0	7969.2

Supplementary Table S3. SE-HPLC peak percentages for EO25 DS formulations (agitated and control). Designations used: Histidine (H); K/Na Phosphate (P); Tris Buffered Saline (TBS); Phosphate Buffered Saline (PBS); pH (in case of phosphate formulations) = 6.5 (65) 7.0 (7), 7.5 (75); Sorbitol (S); Sucrose (Su); EDTA (E), Methionine (M); ExPEC4V DP (C); EO25 DS (25)

Formulation	Pre-peak 1 (%)	Main Peak 1 (%)	Main Peak 2 (%)	Post-peak (%)	Total Area (mAU´s)
Ref. Std.	0.3	17.8	81.9	0.0	9051.3
HSE25 4hr. Agitation	0.1	37.8	62.1	0.0	9523.4
HSE25 Agitation Control	0.1	38.1	61.8	0.0	9100.5
HSE25 4hr. Agitation	0.3	36.6	63.1	0.0	9597.5
HSE25 Agitation Control	0.1	37.5	62.4	0.0	9187.1
HSE25 4hr. Agitation	0.2	36.9	62.9	0.0	9865.8
HS25 Agitation Control	0.1	37.0	62.9	0.0	9670.7
P65SuE25 4hr. Agitation	0.2	37.0	62.8	0.0	9654.7
P65SuE25 Agitation Control	0.1	37.8	62.2	0.0	9284.2
P65SuM25 4hr. Agitation	0.1	40.6	59.3	0.0	9545.5
P65SuM25 Agitation Control	0.1	37.7	62.3	0.0	9304.7
P65Su25 4hr. Agitation	0.2	40.8	59.0	0.0	9503.3
P65Su25 Agitation Control	0.1	37.5	62.4	0.0	9391.2
P7SE25 4hr. Agitation	0.1	38.0	61.9	0.0	9549.7
P7SE25 Agitation Control	0.1	38.2	61.7	0.0	9356.3
P7SM25 4hr. Agitation	0.1	37.8	62.1	0.0	9560.9
P7SM25 Agitation Control	0.1	37.7	62.2	0.0	9435.6
P7S25 4hr. Agitation	0.1	38.9	61.0	0.0	9540.4
P7S25 Agitation Control	0.1	37.5	62.4	0.0	9369.5
P7SuE25 4hr. Agitation	0.1	37.7	62.2	0.0	9439.1
P7SuE25 Agitation Control	0.1	38.0	61.9	0.0	9473.6
P7SuM25 4hr. Agitation	0.1	37.3	62.7	0.0	9649.8
P7SuM25 Agitation Control	0.1	37.7	62.2	0.0	9458.1
P7Su25 4hr. Agitation	0.1	36.5	63.4	0.0	9927.3
P7Su25 Agitation Control	0.1	37.3	62.5	0.0	9820.5
P7SN25 4hr. Agitation	0.1	36.7	63.2	0.0	9492.5
P7SN25 Agitation Control	0.1	37.5	62.4	0.0	9390.6
TBS25 4hr. Agitation	0.1	37.2	62.7	0.0	9147.6
TBS25 Agitation Control	0.1	38.2	61.7	0.0	9474.1
PBS25 4hr. Agitation	34.3	13.1	24.4	28.2	2514.8
PBS25 Agitation Control	0.1	37.9	62.0	0.0	9075.2

Supplementary Table S4. SE-HPLC peak percentages for ExPEC4V DP and ExPEC DS formulations (freeze/thaw). Designations used: Histidine (H); K/Na Phosphate (P); Tris Buffered Saline (TBS); Phosphate Buffered Saline (PBS); pH (in case of phosphate formulations) = 6.5 (65) 7.0 (7), 7.5 (75); Sorbitol (S); Sucrose (Su); EDTA (E), Methionine (M); ExPEC4V DP (C); EO25 DS (25)

Formulation	Pre-peak 1 (%)	Main Peak 1 (%)	Main Peak 2 (%)	Post-peak (%)	Total Area (mAU´s)
Ref. Std.	0.2	17.8	82.0	0.0	8975.7
HSEC	0.0	18.1	81.9	0.0	8637.5
HSMC	0.0	17.9	82.1	0.0	8311.2
HSC	0.0	17.8	82.2	0.0	9012.3
P65SuEC	0.0	18.3	81.7	0.0	8670.8
P65SuMC	0.0	18.0	81.9	0.0	8614.9
P65SuC	0.1	18.0	81.9	0.0	8517.5
P7SEC	0.1	17.9	82.0	0.0	8559.1
P7SMC	0.1	17.7	82.3	0.0	8643.5
P7SC	0.1	17.6	82.3	0.0	8383.1
P7SuEC	0.1	17.4	82.5	0.0	8683.9
P7SuMC	0.1	17.6	82.3	0.0	8182.8
P7SuC	0.1	18.1	81.8	0.0	8699.6
P7SNC	0.1	18.1	81.8	0.0	8719.7
TBSC	0.5	18.9	80.6	0.0	8811.5
PBSC	1.6	20.0	78.4	0.0	7739.0
HSE25	0.1	38.1	61.8	0.0	9059.2
HSM25	0.1	37.8	62.2	0.0	9065.5
HS25	0.1	37.7	62.2	0.0	9411.7
P65SuE25	0.1	38.0	61.9	0.0	9230.5
P65SuM25	0.1	37.8	62.1	0.0	9454.3
P65Su25	0.1	37.9	62.0	0.0	9524.3
P7SE25	0.1	38.1	61.8	0.0	9536.2
P7SM25	0.1	37.7	62.2	0.0	9444.1
P7S25	0.1	37.6	62.3	0.0	9328.0
P7SuE25	0.2	38.1	61.7	0.0	9324.7
P7SuM25	0.1	37.8	62.0	0.0	9390.3
P7Su25	0.1	37.7	62.2	0.0	9593.5
P7SN25	0.1	37.8	62.1	0.0	9379.9
TBS25	0.3	38.8	60.9	0.0	9224.5
PBS25	28.5	20.2	58.7	0.0	882.1

Supplementary Table S5. SE-HPLC peak percentages of ExPEC formulations after 12 weeks at 5°C. Designations used: Histidine (H); K/Na Phosphate (P); Tris Buffered Saline (TBS); Phosphate Buffered Saline (PBS); pH (in case of phosphate formulations) = 6.5 (65) 7.0 (7), 7.5 (75); Sorbitol (S); Sucrose (Su); EDTA (E), Methionine (M); ExPEC4V DP (C); EO25 DS (25)

Formulation	Pre-peak 1 (%)	Main Peak 1 (%)	Main Peak 2 (%)	Post-peak (%)	Total Area (mAU´s)
Ref. Std.	0.1	21.0	78.9	0.0	8703.9
HSEC	0.1	23.7	76.2	0.0	8787.8
HSMC	0.0	23.1	76.9	0.0	8306.3
HSC	0.0	23.7	76.2	0.0	8910.4
P65SuEC	0.0	22.8	77.2	0.0	8330.0
P65SuMC	0.1	22.7	77.2	0.0	8706.2
P65SuC	0.1	23.0	76.9	0.0	8256.1
P7SEC	0.1	24.0	76.0	0.0	8688.0
P7SMC	0.1	23.7	76.2	0.0	8816.8
P7SC	0.1	24.2	75.8	0.0	8427.8
P7SuEC	0.1	22.8	77.1	0.0	8635.0
P7SuMC	0.1	23.6	76.3	0.0	8131.9
P7SuC	0.1	24.5	75.4	0.0	8398.7
P7SNC	0.1	25.7	74.2	0.0	8838.1
TBSC	0.1	21.5	78.4	0.0	9218.1
PBSC	0.1	22.0	77.9	0.0	8210.6
HSE25	0.1	47.5	52.4	0.0	8962.3
HSM25	0.1	47.3	52.6	0.0	8638.4
HS25	0.1	47.5	52.4	0.0	9151.0
P65SuE25	0.0	48.2	51.7	0.0	9005.4
P65SuM25	0.1	47.8	52.1	0.0	9163.5
P65Su25	0.1	47.9	52.0	0.0	9014.4
P7SE25	0.1	47.3	52.6	0.0	9222.6
P7SM25	0.1	47.0	52.9	0.0	9210.5
P7S25	0.1	47.1	52.8	0.0	9025.5
P7SuE25	0.1	48.1	51.9	0.0	9229.6
P7SuM25	0.1	47.0	53.0	0.0	9164.2
P7Su25	0.1	47.7	52.2	0.0	9246.1
P7SN25	0.1	46.1	53.8	0.0	9246.9
TBS25	0.1	41.1	58.9	0.0	9227.4
PBS25	0.1	41.3	58.6	0.0	8866.3

https://doi.org/10.3390/xxxxx

Supplementary Table S6. SE-HPLC peak percentages of three final ExPEC4V formulations (T=O and Freeze/thaw). Designations used: Histidine (H); K/Na Phosphate (P); Tris Buffered Saline (TBS); Phosphate Buffered Saline (PBS); pH (in case of phosphate formulations) = 6.5 (65) 7.0 (7), 7.5 (75); Sorbitol (S); Sucrose (Su); EDTA (E), Methionine (M); ExPEC4V DP (C); EO25 DS (25). F/T – Freeze/thaw cycle.

**Formulation**	**Vial Orientation**	**No. of F/T**	**Pre-peak 1 (%)**	**Main Peak 1 (%)**	**Main Peak 2 (%)**	**Post-peak (%)**	**Total Area (mAU*s)**
DP Control	NA	NA	1.0	19.0	78.5	1.5	8936.4
P7SuEC	upright	1	0.8	18.8	79.0	1.5	9189.9
		2	0.8	18.8	79.0	1.4	9302.1
		3	0.8	18.8	79.0	1.4	9247.9
P7SuEC	inverted	1	0.8	18.9	78.9	1.4	9276.4
		2	0.8	18.8	79.0	1.4	9304.4
		3	0.7	18.6	79.2	1.5	9208.0
P7SMC	upright	1	0.8	18.9	78.8	1.5	9026.6
		2	0.8	18.8	78.9	1.5	9082.0
		3	0.8	18.9	79.1	1.2	9025.4
P7SMC	inverted	1	0.9	19.0	78.5	1.5	8987.5
		2	0.8	18.9	78.8	1.5	9140.1
		3	0.8	18.9	79.0	1.3	9043.9
TBSC	upright	1	1.5	19.2	78.4	0.9	8947.7
		2	1.3	19.1	78.7	1.0	8776.5
		3	1.4	19.0	78.9	0.8	8660.2
TBSC	inverted	1	1.5	18.9	78.8	0.7	8539.9
		2	1.3	19.0	78.7	1.0	8122.0
		3	1.5	19.0	78.7	0.7	8541.1

Supplementary Table S7. SE-HPLC peak percentages of ExPEC formulations after 4 weeks at 40 °C. Designations used: Histidine (H); K/Na Phosphate (P); Tris Buffered Saline (TBS); Phosphate Buffered Saline (PBS); pH (in case of phosphate formulations) = 6.5 (65) 7.0 (7), 7.5 (75); Sorbitol (S); Sucrose (Su); EDTA (E), Methionine (M); ExPEC4V DP (C); EO25 DS (25)

Formulation	Pre-peak 1 (%)	Main Peak 1 (%)	Main Peak 2 (%)	Post-peak (%)	Total Area (mAU´s)
Ref. Std.	0.2	23.2	76.6	0.0	9093.2
HSEC	0.8	21.2	78.1	0.0	8997.4
HSMC	2.0	20.8	77.2	0.0	7986.9
HSC	0.6	21.1	78.3	0.0	9010.0
P65SuEC	0.5	20.9	78.6	0.0	8597.0
P65SuMC	0.5	21.0	78.5	0.0	8867.6
P65SuC	0.6	21.0	78.5	0.0	8432.4
P7SEC	0.4	20.6	79.0	0.0	8806.7
P7SMC	0.3	20.8	78.9	0.0	8838.5
P7SC	0.4	20.6	79.0	0.0	8603.1
P7SuEC	0.6	20.5	78.9	0.0	8945.0
P7SuMC	0.5	20.4	79.2	0.0	8363.1
P7SuC	0.5	20.5	79.0	0.0	8564.6
P7SNC	1.5	20.4	78.1	0.0	8822.3
TBSC	0.6	21.9	77.5	0.0	9048.6
PBSC	0.5	22.5	77.1	0.0	8361.6
HSE25	0.9	41.2	57.9	0.0	9162.0
HSM25	2.6	40.5	56.9	0.0	8807.0
HS25	0.9	41.4	57.7	0.0	9054.1
P65SuE25	0.9	40.8	58.3	0.0	9274.8
P65SuM25	0.7	40.8	58.6	0.0	9386.0
P65Su25	0.8	40.7	58.5	0.0	9163.3
P7SE25	0.5	40.3	59.1	0.0	9304.4
P7SM25	0.3	40.6	59.1	0.0	9028.6
P7S25	0.5	40.4	59.1	0.0	8984.5
P7SuE25	0.9	40.0	59.0	0.0	9347.0
P7SuM25	0.7	40.1	59.3	0.0	9286.3
P7Su25	0.6	40.2	59.2	0.0	9317.0
P7SN25	1.5	40.0	58.6	0.0	9184.8
TBS25	0.7	41.2	58.1	0.0	9222.2
PBS25	0.7	41.4	57.9	0.0	9123.2

Supplementary Table S8. SE-HPLC peak percentages of three final ExPEC4V formulations after 12 weeks of storage at 40°C. Designations used: Histidine (H); K/Na Phosphate (P); Tris Buffered Saline (TBS); Phosphate Buffered Saline (PBS); pH (in case of phosphate formulations) = 6.5 (65) 7.0 (7), 7.5 (75); Sorbitol (S); Sucrose (Su); EDTA (E), Methionine (M); ExPEC4V DP (C); EO25 DS (25)

Formulation	Vial Orientation	Pre-peak(%)	Main Peak 1 (%)	Main Peak 2 (%)	Post-peak (%)	Total Area (mAU*s)
DP Control		1.0	19.1	78.4	1.5	9374.3
PSuEC-1		4.6	15.9	74.7	4.8	8793.7
PSuEC-2	Upright	4.0	16.1	75.3	4.6	8851.1
PSuEC-3		4.0	16.0	75.4	4.5	8825.0
PSuEC-1		4.5	16.1	74.8	4.7	8957.4
PSuEC-2	Inverted	4.2	16.1	75.1	4.6	8884.9
PSuEC-3		3.9	16.1	75.3	4.7	8959.4
PSMC-1		3.2	16.2	75.9	4.7	8731.4
PSMC-2	Upright	3.3	16.1	75.9	4.7	8738.8
PSMC-3		3.3	16.2	76.1	4.4	8663.3
PSMC-1		3.2	16.2	76.0	4.6	8844.7
PSMC-2	Inverted	3.4	16.1	75.8	4.8	8765.1
PSMC-3		3.3	16.2	75.9	4.6	8774.9
TBSC-1		7.0	15.4	73.9	3.7	8922.3
TBSC-2	Upright	6.9	15.3	74.2	3.6	8751.2
TBSC-3		6.8	15.3	74.2	3.6	8652.7
TBSC-1		6.4	15.5	74.3	3.8	9036.1
TBSC-2	Inverted	6.4	15.5	74.2	3.9	9001.8
TBSC-3		6.3	15.5	74.4	3.8	9000.3

https://doi.org/10.3390/xxxxx

Supplementary Table S9. SE-HPLC Peak areas of Tungsten Compatibility Formulations after 4 weeks at 40°C. Designations used: Histidine (H); K/Na Phosphate (P); Tris Buffered Saline (TBS); Phosphate Buffered Saline (PBS); pH (in case of phosphate formulations) = 6.5 (65) 7.0 (7), 7.5 (75); Sorbitol (S); Sucrose (Su); EDTA (E), Methionine (M); ExPEC4V DP (C); EO25 DS (25)

Formulation	Tungsten Level (ng/mL)	Pre-peak (%)	Main Peak 1 (%)	Main Peak 2 (%)	Post-peak (%)	Total Area (mAU*s)
DP Control	0	1.0	18.9	78.6	1.6	9755.2
P7SuEC-HW	2083	3.5	16.7	78.1	1.7	8615.4
P7SuEC-MW	833	2.8	16.8	78.7	1.7	8689.3
P7SuEC-LW	417	2.4	16.9	78.8	1.9	8804.5
P7SuEC Control	0	1.8	17.1	79.1	2.0	8722.8
P7SMC-HW	2083	3.1	17.0	77.7	2.2	8864.8
P7SMC-MW	833	2.5	17.0	78.3	2.2	8947.4
P7SMC-LW	417	2.2	17.0	78.6	2.1	8987.8
P7SMC Control	0	1.7	17.3	78.9	2.1	8982.3
TBSC-HW	2083	8.8	16.1	73.4	1.8	8772.6
TBSC-MW	833	6.0	16.5	75.4	2.1	8907.3
TBSC-LW	417	5.0	16.7	76.4	2.0	8919.0
TBSC Control	0	3.7	17.1	77.3	1.9	8945.1
P7SuE25-HW	2083	3.7	11.7	82.8	1.7	9096.7
P7SuE25-MW	833	2.9	11.8	83.9	1.4	9263.2
P7SuE25-LW	417	2.5	11.9	84.0	1.7	9258.2
P7SuE25 Control	0	1.8	12.1	84.5	1.6	9441.1
P7SM25-HW	2083	3.9	11.7	82.9	1.5	9294.5
P7SM25-MW	833	3.1	11.8	83.3	1.8	9335.3
P7SM25-LW	417	2.6	12.0	83.5	1.9	9373.8
P7SM25 Control	0	2.0	12.1	84.2	1.7	9395.7
TBS25-HW	2083	7.9	11.2	79.6	1.2	9096.1
TBS25-MW	833	5.9	11.6	81.3	1.2	9169.0
TBS25-LW	417	5.2	11.6	81.9	1.3	9130.6
TBS25 Control	0	4.4	11.8	82.3	1.5	9319.3

https://doi.org/10.3390/xxxxx
